# 25*R*-Inokosterone from *Achyranthes bidentata* Ameliorates Parkinson’s Disease Pathology Predominantly via Nrf2/HO-1 Activation with Coordinated MAOB/GSK-3β Expression Downregulation: An In Vitro and In Silico Study

**DOI:** 10.3390/ijms27104204

**Published:** 2026-05-09

**Authors:** Ding Li, Zhi-Ye Chen, Zi-Yang Peng, Liu-Tian Fan, Li-Xia Wu, Xiu-Kun Ma, Ji-Ming Wu

**Affiliations:** Department of Pharmacy, College of Medicine, Jiaxing University, Jiaxing 314001, China; 00200892@stu.zjxu.edu.cn (D.L.); 00200915@stu.zjxu.edu.cn (Z.-Y.C.); 00213318@stu.zjxu.edu.cn (Z.-Y.P.); 00200895@stu.zjxu.edu.cn (L.-T.F.); 00194349@stu.zjxu.edu.cn (L.-X.W.); 00211893@stu.zjxu.edu.cn (X.-K.M.)

**Keywords:** *A. bidentata*, Parkinson’s disease, 25*R*-inokosterone, MAOB and GSK-3β expression modulation, Nrf2 pathway, neuroprotection

## Abstract

Neurological disorders, particularly Parkinson’s disease (PD), represent a pressing global health challenge with limited disease-modifying therapies. While *Achyranthes bidentata* exhibits neuroprotective potential, its bioactive constituents against PD remain poorly characterized. This study integrated phytochemical isolation and in silico target prediction to identify eight compounds from *A. bidentata*, followed by neuroprotective evaluation in 1-Methyl-4-phenyl-1,2,3,6-tetrahydropyridine (MPTP)-challenged SH-SY5Y cells. Among these, 25*R*-inokosterone significantly downregulated Monoamine oxidase B (MAOB) and Glycogen synthase kinase-3β (GSK-3β) expression and showed superior neuroprotection compared to β-ecdysterone. It markedly restored mitochondrial membrane potential, suppressed Bcl-2-associated X protein (Bax)/Cysteinyl aspartate specific proteinase 3 (caspase-3) apoptotic signaling, and alleviated oxidative stress. Mechanistically, Nuclear factor erythroid 2-related factor 2 (Nrf2)/Heme oxygenase 1 (HO-1) activation was the dominant and indispensable mechanism for neuroprotection, while MAOB/GSK-3β expression downregulation served as an upstream synergistic regulatory event, as evidenced by the abolition of neuroprotection following Nrf2 knockdown in SH-SY5Y cells. These findings identify 25*R*-inokosterone as a promising multi-target natural lead for PD, which exerts antioxidant and anti-apoptotic effects predominantly by activating Nrf2, accompanied by the upstream modulation of MAOB/GSK-3β expression.

## 1. Introduction

Diseases affecting the nervous system, either induced by a primary lesion in situ or the significant impact of other systems in the body, can be classified as central nervous system (CNS) or peripheral nervous system (PNS) disorders [1]. The former is commonly observed in cerebrovascular diseases, neurodegenerative diseases, central nervous system infectious diseases, and hereditary metabolic diseases. The latter is primarily known as polyneuropathy and mononeuropathy induced by trauma or other physical and chemical factors [2,3,4]. CNS diseases, especially neurodegenerative disorders such as Parkinson’s disease (PD) and Alzheimer’s disease (AD), are on the rise among individuals with neurological disorders, making it a pressing issue globally [5,6]. PD is commonly characterized by static tremor, postural instability, and myotonia in limb behavior. Pathological studies provide specific descriptions, highlighting the progressive loss of dopaminergic neurons in the substantia nigra and striatum, continuous reduction in dopamine transmitters, and the generation of α-synuclein (SNCA) in the brain as indicators of PD [7]. Clinical treatment for PD or AD commonly involves drug interventions, aiming to enhance the time-effect of neurotransmitters binding with their receptors. The widespread use of levodopa (which is transformed into dopamine by the dopamine decarboxylase, thereby increasing dopamine content in the brain) and donepezil (which inhibits the activity of central cholinesterase and delays the hydrolysis of acetylcholine in the brain) has indeed improved patient outcomes [8,9]. Unfortunately, the long-term use of these drugs leads to compensatory weakening of neuron cell sensitivity to neurotransmitters, gradually diminishing their efficacy [10,11].

Natural products are widely recognized as valuable sources for discovering and developing effective drugs to treat various diseases. For instance, Huperzine-A, derived from *Huperzia serrata*, is a natural drug used in Alzheimer’s disease therapy [12]. However, there remains a scarcity of effective natural medicines for the treatment of PD in clinical practice. In this study, we sought inspiration from the ancient Chinese medical monograph ‘Miscellaneous Records of Famous Physicians’ (ming-yi-bie-lu, in Chinese) regarding the potential ‘prevention of brain diseases’ efficacy of *A. bidentata*. This record indicated a possible neuroprotective effect of *A. bidentata*, prompting us to conduct a series of studies on its chemical constituents and neuroprotective effects. Research on the chemical composition of *A. bidentata* has revealed the presence of phytosterones, triterpenoids, polysaccharides, and flavonoids [13,14,15]. In this study, we present the isolation and structural elucidation of four ecdysterones, along with three isoflavone compounds and a bisphthalate derivative from *A. bidentata*. After the isolation, we conducted anti-PD studies on these compounds using both in silico and in vitro experimental approaches.

Combining the results from Swiss Target Prediction with DAVID (Database for Annotation, Visualization, and Integrated Discovery) functional annotation, we identified various nodes related to PD, including Glycogen synthase kinase-3β (GSK-3β), Monoamine oxidase B (MAOB), DAT, etc. Notably, GSK-3β and MAOB were found to potentially interact with the majority of the isolated ecdysterone compounds. Subsequent analysis, utilizing the STRING dataset-based protein–protein interactions (PPI) network, enabled us to identify nodes linked to the compounds that are significantly associated with neurodegenerative diseases. Additionally, a cell viability assay, along with molecular docking and pharmacophore modeling on these compounds-linked nodes—ranging in binding energies and fitting scores from high to low—suggested a variety of neuroprotective compounds, with 25*R*-inokosterone identified as the most potentially highly efficient anti-neurodegenerative compound. To validate these findings, we conducted a series of experiments using an 1-Methyl-4-phenyl-1,2,3,6-tetrahydropyridine (MPTP)-induced SH-SY5Y cell cytotoxicity model, along with a siRNA transfection assay, to elucidate the anti-neurodegenerative effects of our isolated compounds and the functional mechanism of 25*R*-inokosterone derived from *A. bidentata*.

## 2. Results

### 2.1. Structural Elucidation

**Figure 1:**
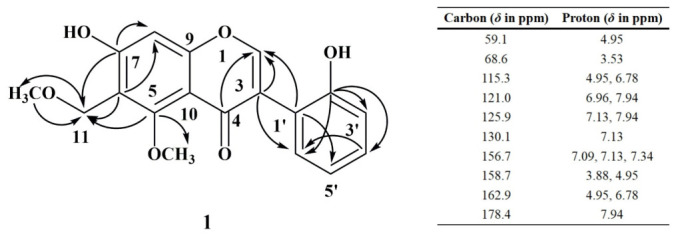
Key HMBC correlations of compound **1**.

Compound **1** was isolated as a light-yellow amorphous powder, and its molecular formula was determined as C_18_H_16_O_6_ with the aid of TOF-MS ([M + Na]^+^ *m*/*z* of 351.0849, calcd. for 351.0845). In the ^1^H-NMR spectrum (400 MHz), it revealed two methyl signals at *δ*_H_ 3.88 (3H, s) and *δ*_H_ 3.53 (3H, s), four ortho-disubstituted benzene hydrogen coupling signals at *δ*_H_ 7.34 (1H, td, *J* = 1.4 Hz and 7.6 Hz), *δ*_H_ 7.13 (1H, dd, *J* = 1.4 Hz and 7.6 Hz), *δ*_H_ 7.09 (1H, brd, *J* = 7.9 Hz), and *δ*_H_ 6.96 (1H, t, *J* = 7.7 Hz), two proton signals at *δ*_H_ 7.94 (1H, s) and *δ*_H_ 6.78 (1H, s), and a set of methylene proton signals at *δ*_H_ 4.95 (2H, s). In the ^13^C-NMR spectrum (100 MHz), three specific carbon signals were observed among the 18 carbon signals, including a carbonyl signal at *δ*_C_ 178.4 and two methoxy group signals at *δ*_C_ 62.9 and *δ*_C_ 59.1. The carbon-proton correlation signal between *δ*_C_ 154.1 and *δ*_H_ 7.94 (1H, s) indicated an isoflavone scaffold for this compound, as confirmed by the HSQC data. On this basis, the observed carbon signal at *δ*_C_ 100.7 in the ^13^C-NMR spectrum was assigned to the C-8 position. Further analysis using the HMBC spectroscopy technique revealed correlations of *δ*_H_ 4.95 with *δ*_C_ 115.3 and 59.1, indicating a linkage of the methylene between one methoxy group and the benzene ring (Figure 1). In combination with the correlations of *δ*_H_ 4.95 with *δ*_C_ 158.7 and 162.9, *δ*_H_ 3.88 with *δ*_C_ 158.7, and *δ*_H_ 6.78 with *δ*_C_ 162.9 but not with *δ*_C_ 158.7, a hydroxyl located at C-7, a -CH_2_-OCH_3_ moiety at C-6, and a methoxy group located at C-6 were confirmed. Additionally, the correlation of *δ*_C_ 156.7 with *δ*_H_ 7.34, 7.09 and 7.13, *δ*_C_ 121.0 with *δ*_H_ 7.94 and 6.96, and *δ*_H_ 7.94 with *δ*_C_ 125.9 and 178.4, indicated a hydroxyl located at C-2′. In combination with the spectroscopy data above, compound **1** was finally recognized as 5-OCH_3_,6-CH_2_-OCH_3_,7-OH,2′-OH-isoflavone, designated as Achyraone A.

Compound **2** was isolated as light-yellow needles, with its molecular formula confirmed as C_19_H_18_O_6_ using TOF-MS ([M + Na]^+^ *m*/*z* of 365.1006, calcd. for 365.1001). The NMR data closely resembled those of compound **1**, except for an additional methyl signal at *δ*_H_ 3.79 (3H, s) in the ^1^H-NMR spectrum (400 MHz) and an extra carbon signal at *δ*_C_ 55.8 in the ^13^C-NMR spectrum (100 MHz). In the HSQC spectra data, the direct proton-carbon correlations of the substituents in ring A exhibited high similarity, except for the newly formed correlations of *δ*_H_ 3.79 with *δ*_C_ 55.8. Taking all aspects mentioned above into consideration, compound **2** was conclusively determined as 5-OCH_3_, 6-CH_2_-OCH_3_,7-OH,2′-OCH_3_-isoflavone, and designated as Achyraone B.

**Figure 2:**
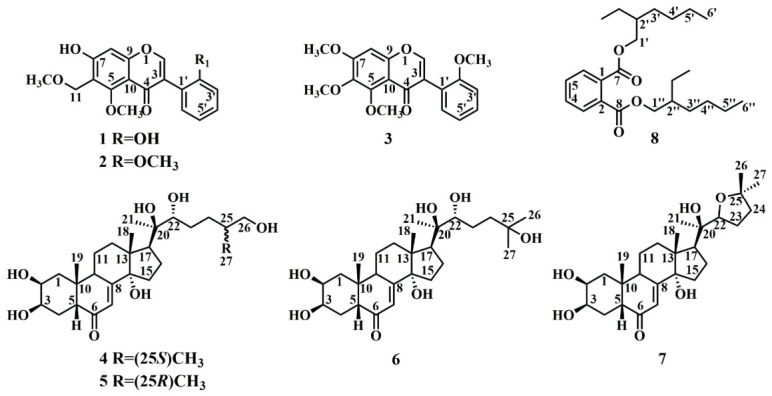
Chemical structures of eight compounds isolated from *A. bidentata*.

The absolute configurations of compounds **3**–**8** (Figure 2), including 5,6,7,2′-tetramethoxyisoflavone (**3**), 25*S*-inokosterone (**4**), 25*R*-inokosterone (**5**), β-ecdysterone (**6**), stachysterone D (**7**), and bis(2-ethylhexyl)-phthalate (**8**), were elucidated by comparison of their ^1^H- and ^13^C-NMR data with literature values [16,17,18]. Together with compounds **1** and **2**, the complete ^1^H- and ^13^C-NMR chemical shift assignments for all compounds are summarized in Table 1 and Table 2.

### 2.2. GSK-3β and MAOB Are Likely to Be the Binding Targets for the A. bidentata Compounds to Exert the Anti-Neurodegeneration Efficacy

**Figure 3:**
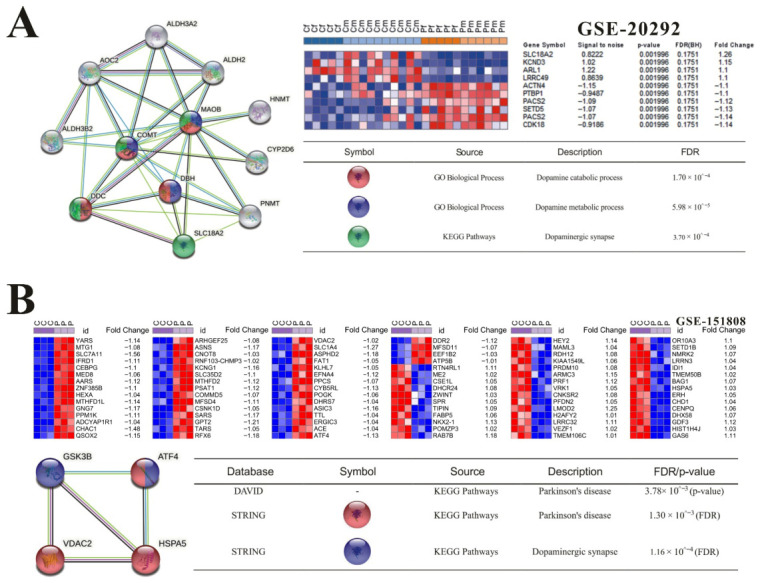
(**A**) Identification of top-ranked DEGs from the substantia nigra of PD patients (GSE-20292) and their correlation with Monoamine oxidase B (MAOB)—a potential target of isolated ecdysterone compounds. Left: PPI network constructed via STRING showing interactions between MAOB and dopamine metabolism-related genes; **Upper right**: Heatmap and statistical metrics of DEGs; **Lower right**: GO and KEGG pathway enrichment analysis (FDR values indicated); (**B**) Identification of DEGs from MPP^+^-treated SH-SY5Y cells (GSE-151808) and their correlation with Glycogen synthase kinase-3β (GSK-3β)—a potential target of isolated ecdysterone compounds. **Upper panel**: Heatmaps of top-ranked DEGs with fold change values; **Lower left**: STRING-based PPI network showing GSK-3β interactions with ER stress-related proteins (ATF4, HSPA5, VDAC2); **Lower right**: KEGG pathway enrichment analysis with FDR/*p*-values indicated.

Functional enrichment analysis via DAVID 6.8 identified significant enrichment of neurodegeneration-associated pathways among targets of *A. bidentata* bioactive compounds, particularly ecdysterones and polyphenol derivatives [16,19,20,21]. The top-ranked GO terms (such as GO-BP: synaptic transmission, dopaminergic, and GO-MF: tau-protein kinase activity) showed significant enrichment and contained key nodes related to PD/AD pathogenesis (Table 3). Notably, our bioinformatics analysis revealed that the isolated ecdysterones exhibited significant regulatory correlations with GSK-3β and MAOB (*p* < 1 × 10^−5^), both of which are well-established therapeutic targets for Parkinson’s disease. Integration of differentially expressed genes from GSE20292 (PD patient-derived) datasets via STRING PPI network analysis uncovered a critical interaction module involving SLC18A2, MAOB, COMT, DDC, and DBH (Figure 3A). This module showed significant enrichment in the KEGG Parkinson’s disease pathway. Besides that, correlation analysis also confirmed physical interactions between GSK-3β and endoplasmic reticulum (ER) stress mediators HSPA5, VDAC2, and ATF4 in GSE151808 (MPP^+^-treated SH-SY5Y cells), revealing a potential involvement of the unfolded protein response in Parkinson’s disease pathogenesis (Figure 3B).

Emerging evidence highlights GSK-3β as a critical kinase in neurodegeneration. While its hyperactivity promotes tau phosphorylation at multiple sites (Ser199/136/413) contributing to the pathogenesis of AD [22,23], it similarly drives Parkinsonian pathology through α-synuclein phosphorylation at Ser129 [24]. Notably, dysregulated GSK-3β activity disrupts crosstalk between signaling pathways in neurodegeneration, concomitantly driving mitochondrial dysfunction, oxidative stress, and ER stress. For instance, GSK-3β-induced phosphorylation of Nuclear factor erythroid 2-related factor 2 (Nrf2) at Ser335/Ser338 promotes its degradation, thereby suppressing antioxidant responses and amplifying oxidative damage [25]. Mechanistically, GSK-3β activation is triggered by calpain-dependent proteolytic cleavage of its C-terminal or N-terminal domains [26]. This activation cascade upregulates the pro-apoptotic BH3-only protein Bim, which subsequently activates Bcl-2-associated X protein (Bax), caspase-9, and Cysteinyl aspartate specific proteinase 3 (caspase-3), culminating in mitochondrial apoptosis [27].

Previously, MAOB was thought to drive PD pathogenesis by hydrolyzing dopamine neurotransmitters [28]. However, contemporary studies reveal that dopamine metabolism in the substantia nigra is predominantly mediated by MAOA. Despite this, MAOB remains therapeutically significant in PD. Beyond its role in degrading neurotransmitters (including MPTP), MAOB activation promotes aberrant reactivity in astrocytes. Reactive astrocytes secrete GABA, which suppresses the viability of dopaminergic neurons. This process also elevates H_2_O_2_ levels in the substantia nigra, contributing to neuronal death [29]. Additional research demonstrates that MAOB activation induces α-synuclein aggregation in SH-SY5Y cells. Notably, MAOB inhibitors like selegiline facilitate the extracellular release of detergent-insoluble α-synuclein from the cytoplasm of these cells [30]. In MPTP-induced rodent models of PD, MAOB also serves as a critical enzyme converting non-toxic MPTP into the neurotoxic metabolite MPP^+^ [31].

Online target prediction analyses have identified significant binding potential between *A. bidentata*-derived compounds and both GSK-3β and MAOB. Notably, compounds specifically isolated in the current investigation exhibit potential regulatory profiles against these targets. If validated experimentally, their precise mechanisms of action could underpin novel therapeutic strategies for neurodegenerative disorders.

### 2.3. In Silico Determination of Potentially Effective MAOB and GSK-3β Binding Ligands from the Isolated A. bidentata Compounds

**Figure 4:**
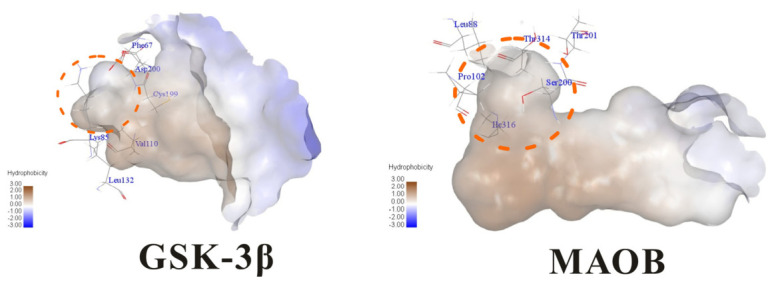
Structural characterization of the binding pockets of GSK-3β and MAOB. Left: GSK-3β binding pocket showing the hydrophobic cavity comprising Phe67, Lys85, Val110, Leu132, Cys199, and Asp200; Right: MAOB binding pocket displaying the hydrophobic pocket surrounded by Leu88, Pro102, Ser200, Thr201, Thr314, and Ile316. Surface hydrophobicity is color-coded from brown (hydrophobic) to blue (hydrophilic).

Molecular docking studies were performed to evaluate the binding potential and expression regulatory effects of several known expression modulators on GSK-3β and MAOB [32,33,34,35,36,37,38,39,40,41,42,43]. Based on a comprehensive analysis of binding modes and free energy calculations, the top two ligands for each target were prioritized. Structural analysis revealed a hydrophobic cavity formed by Phe67, Lys85, Val110, Leu132, Cys199, and Asp200 within the GSK-3β binding site. Ligands featuring hydrophobic moieties embedded in this cavity exhibited significantly enhanced absolute binding energy values. Similarly, a distinct hydrophobic pocket surrounded by Leu88, Pro102, Ser200, Thr201, Thr314, and Ile361 in MAOB demonstrated analogous binding characteristics (Figure 4). Further investigation of all *Achyranthes bidentata*-derived compounds targeting GSK-3β identified ecdysterones as dominant binders, with all top five ranked candidates belonging to this class. Consistent with reference compounds, these ecdysterones showed superior binding affinity when optimally occupying the GSK-3β hydrophobic cavity, correlating with higher binding energy values compared to other ecdysterone analogs. This structure-activity relationship was also validated for MAOB-bound complexes (Table 4). Notably, three ecdysterones—20*R*,22*R*-2β,3β,20,22,26-pentahydroxy-cholestan-7,12-dien-6-one, β-ecdysterone, and 25*R*-inokosterone—demonstrated dual-target binding capacity against both GSK-3β and MAOB. Among these, β-ecdysterone and 25*R*-inokosterone represent our isolates characterized in this study.

**Figure 5:**
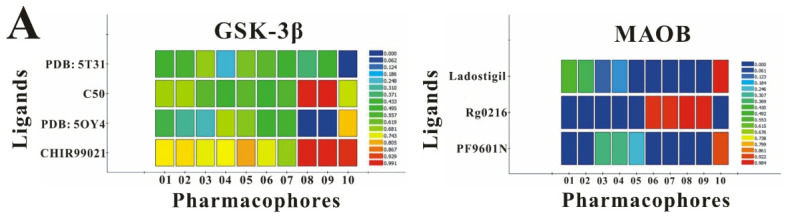

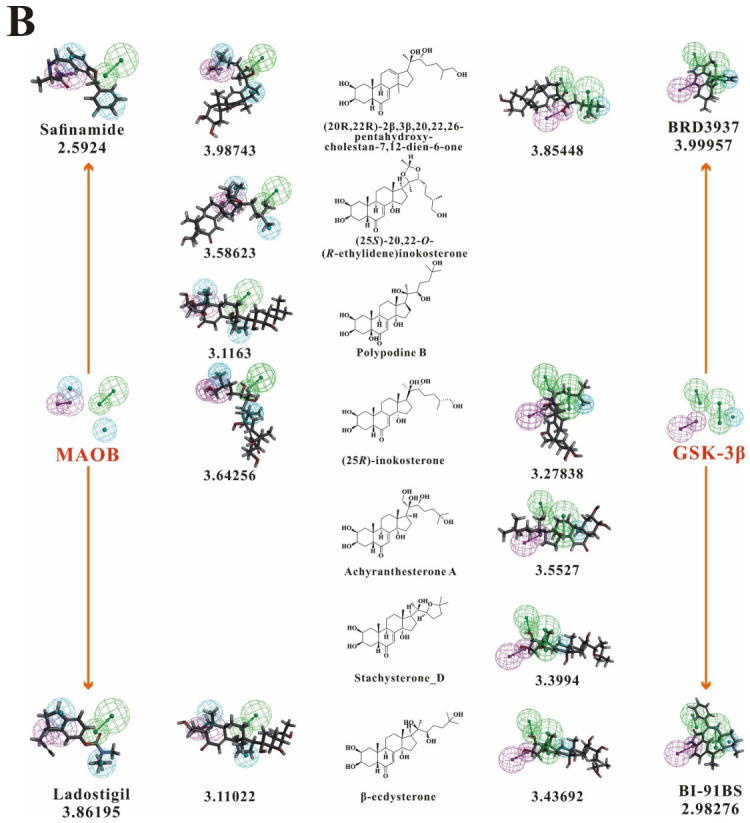
Pharmacophore-based virtual screening of ecdysterone compounds as dual GSK-3β/MAOB expression modulators. (**A**) Validation of pharmacophore models using known reference ligands: GSK-3β inhibitors (**left**) and MAOB inhibitors (**right**). Pharmacophore 5 (P5) and Pharmacophore 10 (P10) exhibited optimal performance for GSK-3β and MAOB, respectively. (**B**) Three-dimensional pharmacophore mapping of reference modulators and isolated ecdysterones, demonstrating dual-target compliance of 20*R*,22*R*-2β,3β,20,22,26-pentahydroxy-cholestan-7,12-dien-6-one, 25*R*-inokosterone, and β-ecdysterone with both P5 and P10 models (fitness scores indicated).

To corroborate our docking results and evaluate the expression regulatory potential of *A. bidentata* compounds on GSK-3β and MAOB, we conducted ligand-based pharmacophore modeling. The training set for each target was constructed using structurally diverse known active ligands, while the test set was generated via a “diverse scaffold” partitioning strategy (Table A1). Pharmacophore models exhibiting ≥4 functional features were prioritized for further analysis. Among these, Pharmacophore 5 (P5) for GSK-3β—comprising one hydrogen bond donor, two hydrogen bond acceptors, and one hydrophobic center—and Pharmacophore 10 (P10) for MAOB—containing one hydrogen bond donor, one hydrogen bond acceptor, and two hydrophobic centers—demonstrated optimal validation metrics in rigorous testing (Table 5, Figure 5A). These validated models (P5/P10) were subsequently employed to screen the *A. bidentata* compound library. Under strict inclusion criteria permitting no omitted pharmacophoric features, only ecdysterones successfully mapped onto P5 or P10 with significant fitting scores (Figure 5B). Notably, three ecdysterones—20*R*,22*R*-2β,3β,20,22,26-pentahydroxy-cholestan-7,12-dien-6-one, β-ecdysterone, and 25*R*-inokosterone—exhibited dual-target compliance, aligning with both P5 and P10 specifications. This dual-pharmacophore adherence strongly suggests potential regulatory effects on GSK-3β and MAOB protein expression. Consistent with molecular docking outcomes, β-ecdysterone and 25*R*-inokosterone emerged as experimentally isolated compounds possessing superior binding profiles. Crucially, 25*R*-inokosterone displayed enhanced performance in molecular modeling, suggesting preferential in vitro anti-Parkinsonian efficacy compared to β-ecdysterone. This computational advantage positions 25*R*-inokosterone as a promising candidate for future biological evaluation.

### 2.4. Validation of Pharmacophore Models and Binding Poses Stability

**Figure 6:**
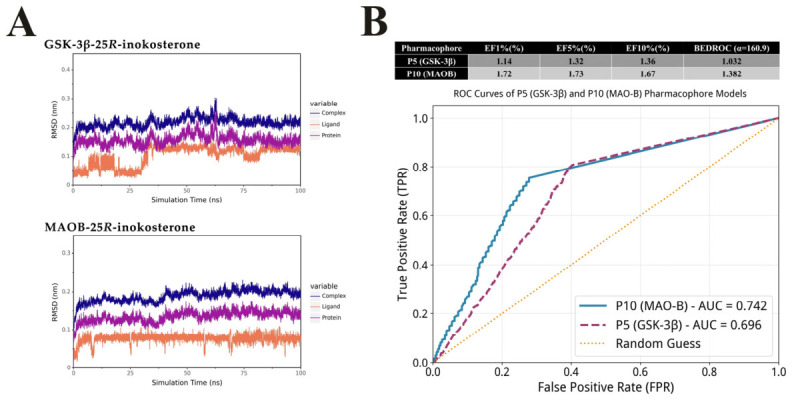
Validation of binding poses stability via molecular dynamics simulation (**A**) and pharmacophore models via active ligands and decoy validations (**B**).

According to the 100 ns molecular dynamics simulation results, the complexes of GSK-3β and MAOB with 25*R*-inokosterone both reached equilibrium after an initial equilibration phase. The root-mean-square deviation (RMSD) analysis revealed that both systems remained stable throughout the simulation. After the initial equilibration phase, the protein backbone RMSD of both systems stabilized at approximately 0.15–0.20 nm, with no significant conformational changes observed. For the ligand 25R-inokosterone, the RMSD values converged to a narrow range of 0.05–0.15 nm in both systems, which was well below the 0.2 nm threshold commonly used to indicate stable binding. Specifically, the ligand RMSD in the MAOB complex was even smaller, fluctuating around 0.05–0.10 nm, demonstrating the extremely high binding stability (Figure 6A). These results confirmed that despite the flexibility of 25R-inokosterone, the binding poses predicted by molecular docking were dynamically stable, validating the reliability of the docking results.

To evaluate the effectiveness of the pharmacophore models, we first sorted the combined set of decoy molecules and active molecules in descending order according to their Fit Value with the pharmacophore, and then systematically evaluated the screening performance of the two pharmacophore models. For the MAOB model (P10), which was validated using a dataset of 1352 active ligands and 2080 decoys, we identified 23 active molecules among the top 1% ranked molecules (34 in total), 117 active molecules among the top 5% ranked molecules, and 226 active molecules among the top 10% ranked molecules. The corresponding enrichment factors were 1.72, 1.73, and 1.67, with an ROC-AUC of 0.742 and a BEDROC value of 1.382. For the GSK-3β model (P5), which was validated using a dataset of 1389 active ligands and 2162 decoys, we found 16 active molecules among the top 1% ranked molecules (36 in total), 92 active molecules among the top 5% ranked molecules, and 189 active molecules among the top 10% ranked molecules. The corresponding enrichment factors were 1.14, 1.32, and 1.36, with an ROC-AUC of 0.696 and a BEDROC value of 1.032 (Figure 6B). These results demonstrated that both pharmacophore models exhibited good discriminative ability and favorable early enrichment efficiency, confirming their reliable screening performance.

### 2.5. Assigning 25R-Inokosterone as the Representative Compound to Conduct the Neuroprotection Assays

**Figure 7:**
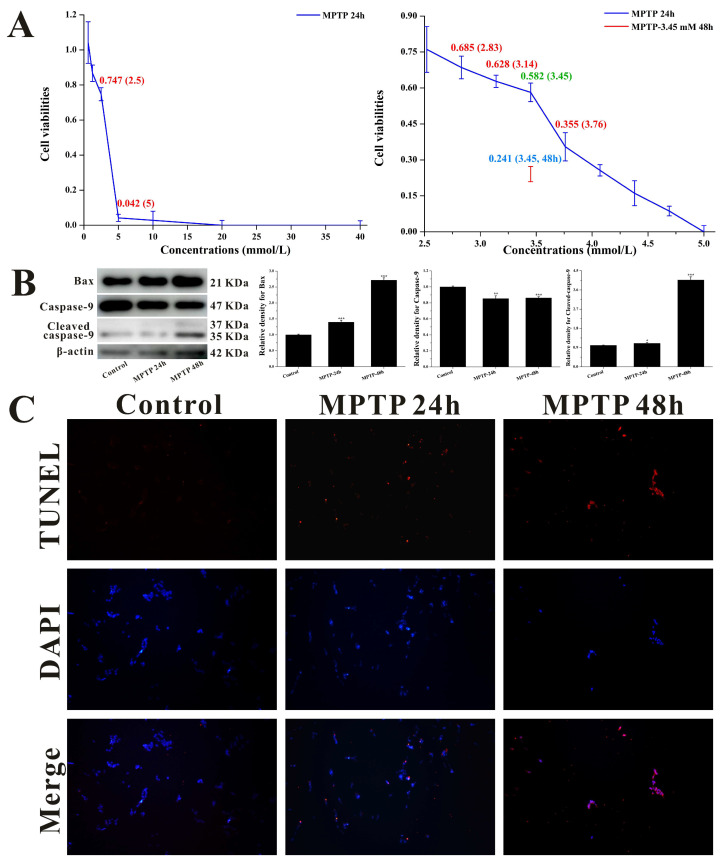
Characterization of 1-Methyl-4-phenyl-1,2,3,6-tetrahydropyridine (MPTP)-induced neurotoxicity in SH-SY5Y cells. (**A**) Cell viability assays identifying 3.45 mmol/L MPTP for 24 h as the optimal modeling condition, with 48 h treatment excluded due to excessive cytotoxicity. (**B**) Western blotting quantification demonstrating MPTP-triggered mitochondrial apoptosis via Bcl-2-associated X protein (Bax) upregulation and caspase-9 activation. Data are expressed as mean ± SD. * *p* < 0.05, ** *p* < 0.01, *** *p* < 0.001 versus control. (**C**) TUNEL staining visualizing time-dependent DNA fragmentation, corroborating apoptotic cell death (100×).

To establish a cell neurotoxicity model, we employed MPTP (rather than its direct metabolite MPP^+^) for inducing cytotoxicity in SH-SY5Y cells. This methodological choice was predicated on the well-established metabolic pathway wherein MAOB-mediated conversion of MPTP generates MPP^+^, thereby enabling indirect assessment of potential MAOB expression regulatory effects exerted by β-ecdysterone and 25*R*-inokosterone. As shown in Figure 7A, dose-dependent reductions in cellular viability were observed 24 h post-MPTP administration. Through systematic optimization, we identified 3.45 mM MPTP as the optimal challenge concentration, producing 58.2% cell viability—a threshold satisfying our predefined criteria (45–65% survival) for establishing a valid injury model. Under these optimized conditions, immunoblotting analyses revealed significant upregulation of pro-apoptotic Bax and cleaved caspase-9 expression (Figure 7B), corroborated by TUNEL assay demonstrating enhanced DNA fragmentation indicative of apoptotic progression (Figure 7C). Notably, while prolonged 48 h MPTP exposure induced more pronounced elevations in apoptotic biomarkers, the resulting severe cytotoxicity (24.1% viability) rendered this condition unsuitable for subsequent pharmacological investigations.

**Figure 8:**
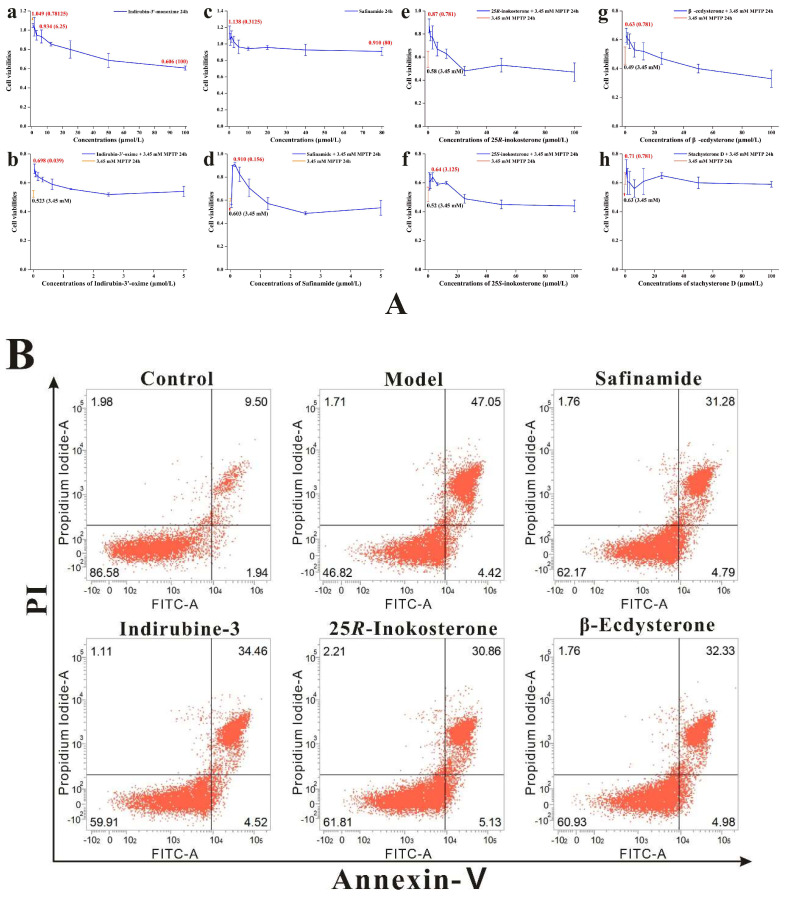
(**A**) Neuroprotective effects of reference inhibitors and ecdysterones against MPTP-induced SH-SY5Y cell injury. (**a**–**d**) GSK-3β inhibitor indirubin-3′ and MAOB inhibitor safinamide significantly improved cell viability at optimal concentrations; (**e**–**h**) Among four isolated ecdysterones, 25*R*-inokosterone exhibited the most potent neuroprotection at the low concentration (0.78 μM), supporting its potential as a dual-target expression modulator of GSK-3β and MAOB. (**B**) Flow cytometric analysis of apoptosis by Annexin V-FITC/PI dual staining. 25*R*-inokosterone (0.78 μM) significantly attenuated MPTP-induced apoptosis in SH-SY5Y cells, reducing late apoptotic cells to 30.86%, which was superior to β-ecdysterone (32.33%), confirming its optimal anti-apoptotic activity.

The neuroprotective potential of all isolated compounds was evaluated using the CCK-8 assay in the validated MPTP-induced SH-SY5Y cell injury model. Preliminary cytotoxicity assessment revealed that neither ecdysterones nor the bisphthalate derivative exhibited significant toxicity across tested concentrations when administered independently (Table 6). However, phenolic derivatives (specifically isoflavones) demonstrated a reduction in cell viability at 100 μM, mirroring the cytotoxic profile observed with indirubin-3′ (GSK-3β inhibitor positive control), while safinamide (MAOB inhibitor) maintained cellular integrity under identical conditions. Subsequent co-treatment experiments demonstrated that pre-incubation with specific non-toxic concentrations of ecdysterones or reference inhibitors significantly attenuated MPTP-induced cytotoxicity, as evidenced by elevated cell viability compared to MPTP-only controls (Table 6, Figure 8A). Notably, 25*R*-inokosterone exhibited the most pronounced recovery of cell viability with an EC_50_ of 0.39 μM (defined as the concentration producing 50% of the maximal neuroprotective effect against MPTP-induced injury), consistent with flow cytometry analyses showing concomitant reduction in apoptosis rates (Figure 8B). In contrast, neither phenolic derivatives nor the bisphthalate compound conferred protection against MPTP-mediated damage, regardless of concentration. Integration of computational simulation data with empirical findings identified 25*R*-inokosterone as the optimal candidate for further investigation, owing to its superior performance in both molecular docking/pharmacophore modeling and functional validation assays. This dual confirmation positions compound **5** as a promising lead for subsequent mechanistic studies on neurodegenerative pathology.

### 2.6. 25R-Inokosterone Restored GSH and Dopamine Levels with Concomitant Reduction in LDH Leakage in SH-SY5Y Cells

**Figure 9:**
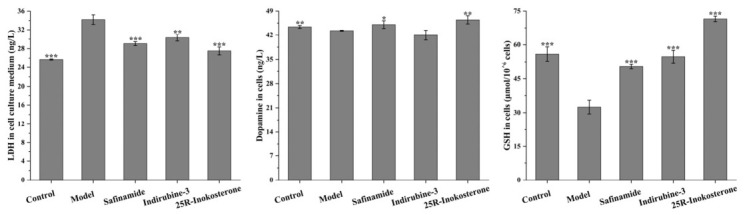
25*R*-inokosterone restores cellular homeostasis in MPTP-injured dopaminergic neurons. ELISA analyses demonstrate that 0.78 μM 25*R*-inokosterone significantly reduced LDH leakage (**left**), preserved dopamine content (**middle**), and enhanced GSH reserves (**right**) in SH-SY5Y cells. Compared with the positive control group, these effects implicate dual-target expression downregulation of MAOB and GSK-3β as the mechanistic basis for 25*R*-inokosterone’s cytoprotective activity. Data are expressed as mean ± SD. * *p* < 0.05, ** *p* < 0.01, *** *p* < 0.001 versus model.

ELISA analyses revealed that 0.78 μM 25*R*-inokosterone significantly attenuated MPTP-induced elevation of extracellular LDH levels, with an efficacy comparable to that observed in the safinamide and indirubine-3′ treatment groups. Subsequent biochemical profiling demonstrated that this compound effectively restored intracellular dopamine concentrations and GSH reserves in damaged cells. Notably, these restorative effects approached physiological homeostasis levels observed in the control group, suggesting the significant cellular protective effects (Figure 9). The observed correlation between LDH suppression, neurotransmitter preservation, and antioxidant replenishment suggests that the modulation of MAOB and GSK-3β protein expression is closely associated with the cytoprotective activity of 25*R*-inokosterone.

### 2.7. Reduction in MAOB and GSK-3β Protein Levels by 25R-Inokosterone Was Associated with the Alleviation of MPTP-Induced Cytotoxicity in SH-SY5Y Cells

**Figure 10:**
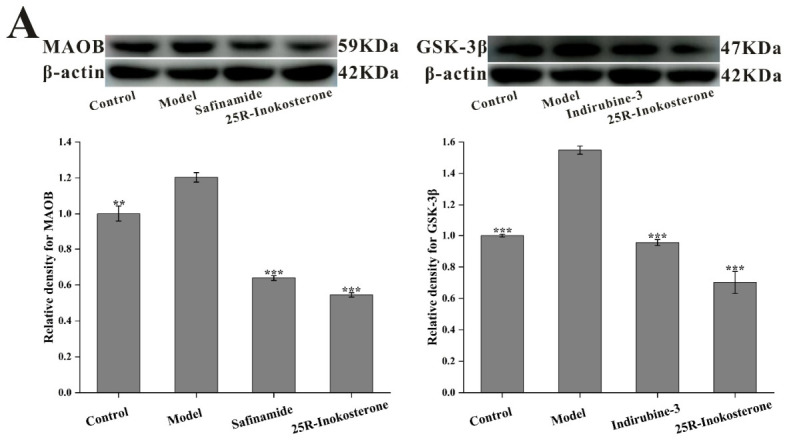

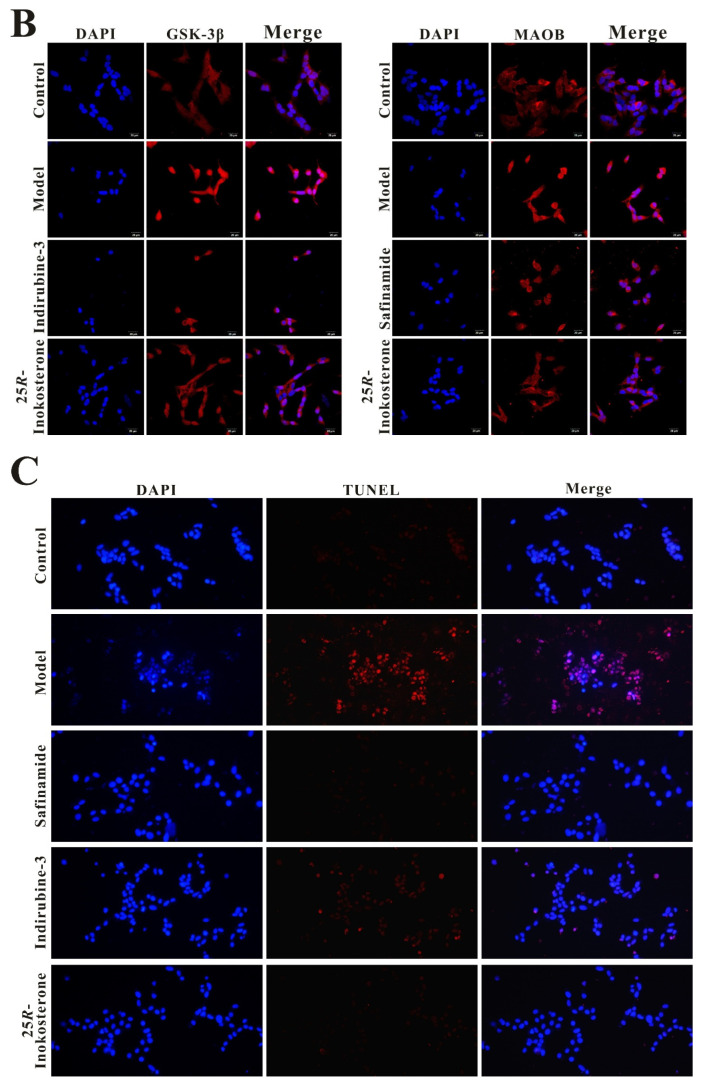
Molecular validation of dual-target expression regulation by 25*R*-inokosterone. (**A**) Western blots showing that 25*R*-inokosterone significantly downregulated MPTP-induced upregulation of both MAOB and GSK-3β, mirroring the effects of reference inhibitors safinamide and indirubin-3′. Data are expressed as mean ± SD. ** *p* < 0.01, *** *p* < 0.001 versus model. (**B**) Immunofluorescence images revealing diminished fluorescence intensity of GSK-3β and MAOB in 25*R*-inokosterone-treated cells (600×). (**C**) TUNEL assay visualizing the anti-apoptotic efficacy of 25*R*-inokosterone through marked reduction in DNA strand breaks compared to the MPTP model (200×).

Building upon observed improvements in cellular viability and biochemical parameters, we investigated 25*R*-inokosterone’s capacity to modulate MAOB and GSK-3β expression in MPTP-injured SH-SY5Y cells. Western blot analyses revealed downregulation of both MAOB and GSK-3β following 25*R*-inokosterone treatment, mirroring the expression regulatory profiles of safinamide and indirubin-3′ (Figure 10A). These molecular findings were corroborated by immunofluorescence microscopy, which demonstrated considerably reduced signal intensity for both target proteins in treated groups relative to the model group (Figure 10B). Critically, TUNEL staining revealed an obvious reduction in DNA fragmentation among 25*R*-inokosterone-treated cells compared to lesioned controls. This anti-apoptotic efficacy was comparable to positive controls treated with safinamide or indirubine-3′ (Figure 10C). Mechanistically, the observed neuroprotection is closely correlated with the modulation of MAOB and GSK-3β protein expression.

### 2.8. 25R-Inokosterone Rescued MPTP-Induced SH-SY5Y Cell Lesion, MMP Loss and ROS Elevation

**Figure 11:**
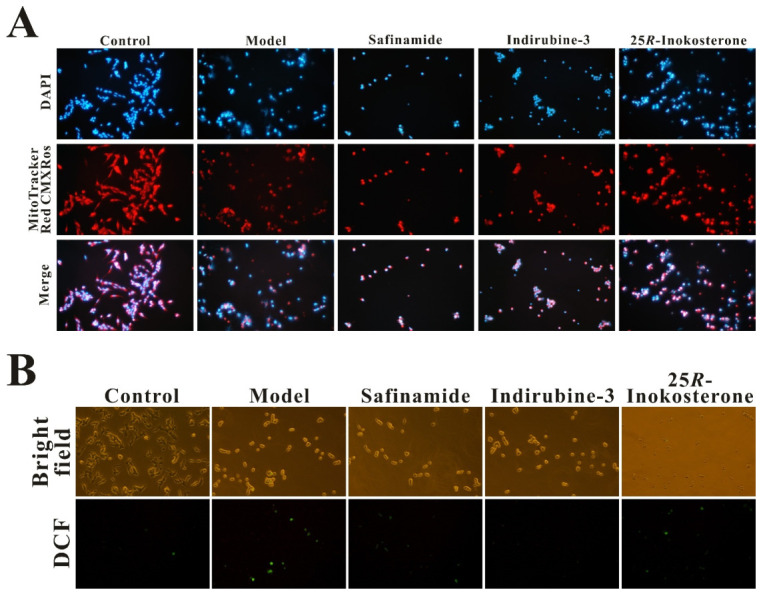

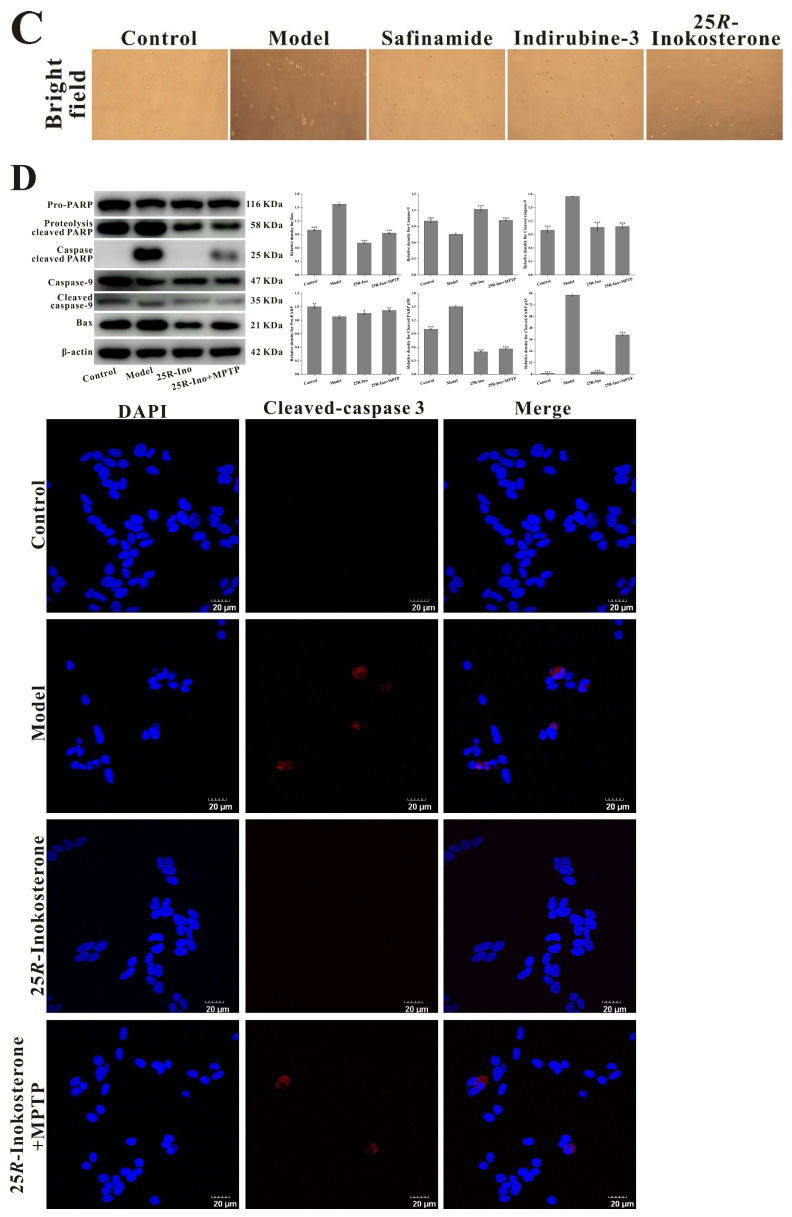
25*R*-inokosterone mitigates MPTP-induced mitochondrial dysfunction, oxidative stress, and apoptotic cascades. (**A**) 25*R*-inokosterone significantly restored MMP loss in MPTP-injured SH-SY5Y cells (200×). (**B**) 25*R*-inokosterone showed effective ROS-scavenging activity (200×). (**C**) 25*R*-inokosterone improved MPTP-induced cell damage and morphological abnormalities to a considerable extent (200×). (**D**) Quantitative analysis of apoptotic signaling reveals that 25*R*-inokosterone downregulates Bax, cleaved caspase-9, and cleaved PARP expression (data are expressed as mean ± SD. * *p* < 0.05, ** *p* < 0.01, *** *p* < 0.001 versus model), with concomitant reduction in cleaved Cysteinyl aspartate specific proteinase 3 (caspase-3) immunoreactivity as evidenced by decreased fluorescence intensity following treatment (600×).

In the supplemented cell morphology assay depicted in Figure 11A, Mito-tracker red CMXRos staining (Beyotime C1071S) demonstrated intense red fluorescence in the control group, indicating robust MMP. However, following 24 h treatment with MPTP in SH-SY5Y cells, fluorescence intensity significantly diminished compared to controls, suggesting induction of mitochondrial dysfunction. Notably, pretreatment with 25*R*-inokosterone, safinamide, or indirubin-3′ markedly restored MMP-associated fluorescence, with 25*R*-inokosterone exhibiting the most pronounced protective effect on mitochondrial integrity. Consistent with these findings, DCFH-DA staining (Figure 11B) confirmed that 25*R*-inokosterone effectively attenuated MPTP-induced ROS accumulation, as evidenced by the stark contrast between elevated fluorescence in the model group and reduced signal in the 25*R*-inokosterone treatment group. This antioxidant capacity likely correlates with 25*R*-inokosterone’s ability to upregulate Nrf2-mediated antioxidant response elements, thereby enhancing cellular redox homeostasis.

Bright-field microscopy revealed pronounced differences in cellular morphology between the MPTP-induced model group and the 25*R*-inokosterone-treated group. Specifically, the model group exhibited a higher incidence of cell floating and shrinkage (hallmarks of apoptotic degeneration) compared to the treatment group (Figure 11C). This morphological preservation aligned with Western blot analyses demonstrating 25*R*-inokosterone’s capacity to mitigate MPTP-induced apoptotic signaling. The compound effectively suppressed MPTP-triggered upregulation of pro-apoptotic factors Bax, caspase-9, and caspase-3, while reversing the downregulation of anti-apoptotic PARP in SH-SY5Y cells (Figure 11D). Collectively, these molecular events (coupled with 25*R*-inokosterone’s dual expression downregulation of MAOB and GSK-3β) provide converging evidence for its neuroprotective mechanism. By simultaneously targeting mitochondrial dysfunction, oxidative stress, and apoptotic cascades, while modulating MAOB and GSK-3β expression, 25*R*-inokosterone demonstrates multifaceted efficacy in mitigating MPTP-induced neuronal injury.

### 2.9. Knockdown of Nrf2 Interfered 25R-Inokosteron Mediated Neuroprotection on SH-SY5Y Cells

**Figure 12:**
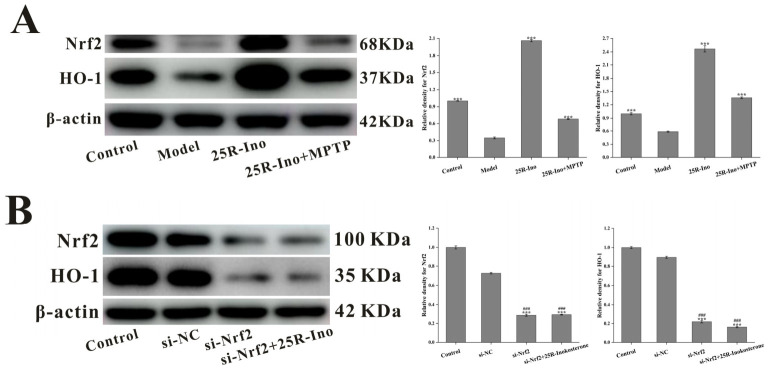

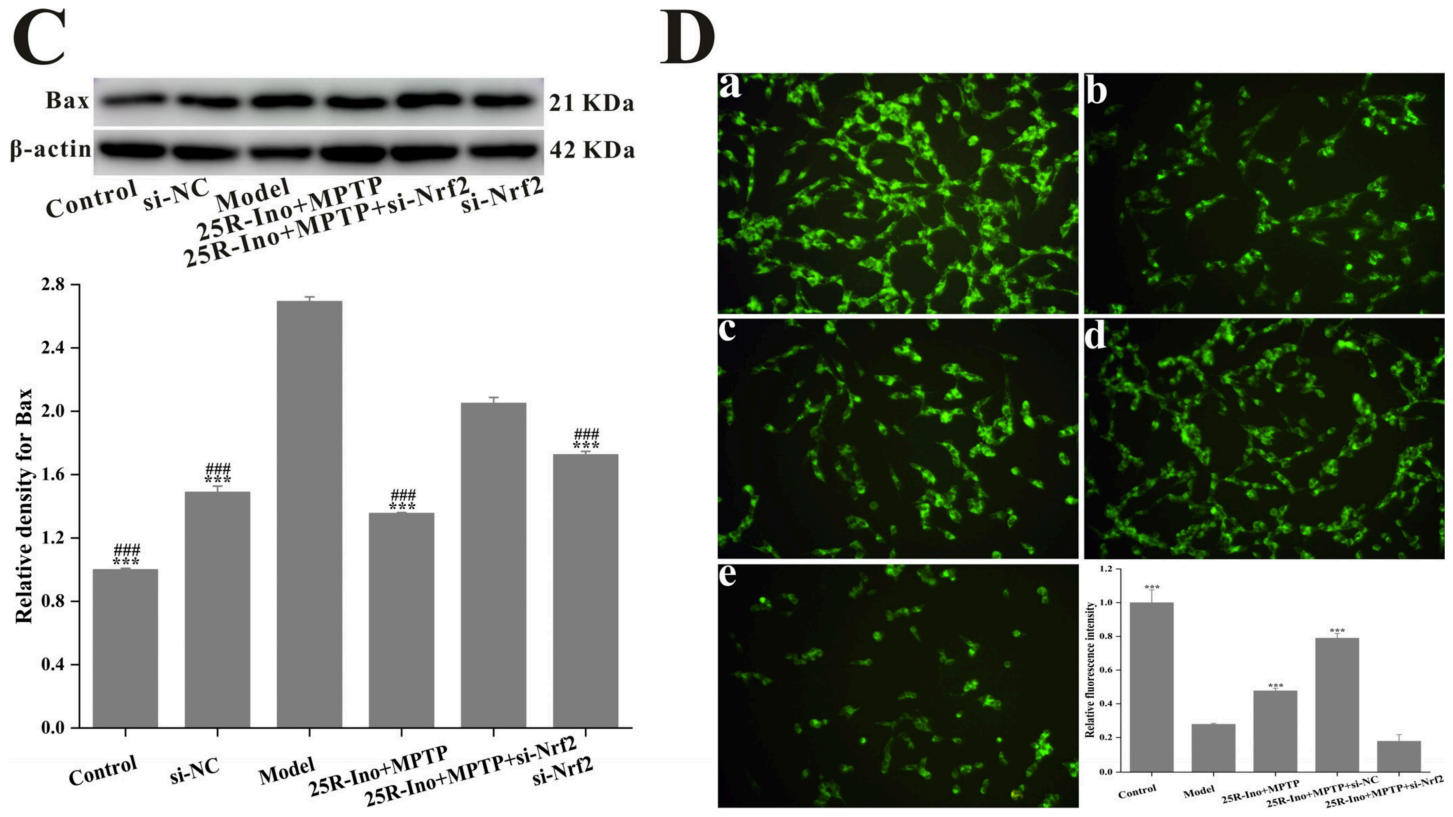
Nuclear factor erythroid 2-related factor 2 (Nrf2)/Heme oxygenase 1 (HO-1) signaling constitutes a critical mechanism for 25R-inokosterone-mediated neuroprotection. (**A**) 25*R*-inokosterone upregulates Nrf2/HO-1 pathway expression and rescues MPTP-induced downregulation of Nrf2/HO-1 signaling. Data are expressed as mean ± SD. *** *p* < 0.001 versus model. (**B**) Nrf2 knockdown by siRNA attenuates downstream HO-1 expression and abrogates 25*R*-inokosterone-mediated upregulation of the Nrf2/HO-1 pathway. Data are expressed as mean ± SD. *** *p* < 0.001 versus control. ### *p* < 0.001 versus si-NC. (**C**) Nrf2 silencing severely impairs the regulatory effect of 25*R*-inokosterone on mitochondria-associated apoptotic protein expression in MPTP-challenged SH-SY5Y cells. Data are expressed as mean ± SD. *** *p* < 0.001 versus model. ### *p* < 0.001 versus 25*R*-ino+MPTP+si-Nrf2. (**D**) Nrf2 knockdown disrupts the protective efficacy of 25*R*-inokosterone against MPTP-induced MMP collapse in SH-SY5Y cells (200×) (**a**–**e**). Data are expressed as mean ± SD. *** *p* < 0.001 versus model.

**Figure 13:**
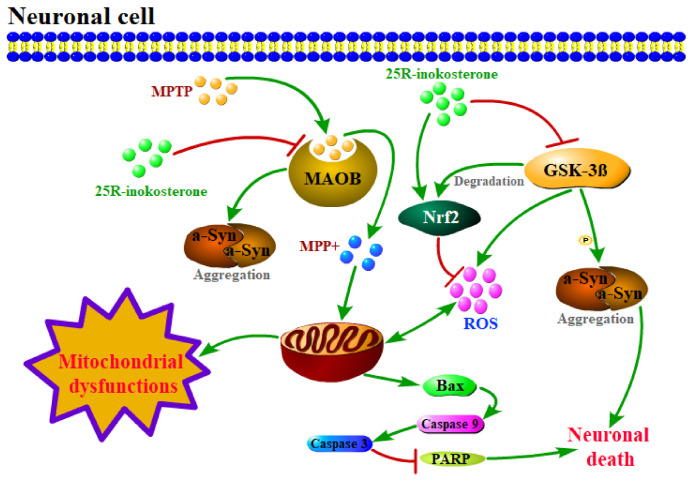
25*R*-inokosterone exerts neuroprotection mainly via Nrf2/HO-1 activation, with coordinated modulation of MAOB and GSK-3β expression, coupled with Nrf2 activation and subsequent HO-1 upregulation, thereby ameliorating ROS-mediated mitochondrial apoptosis. This polypharmacological intervention alleviates mitochondrial dysfunction and prevents neuronal death.

The Nrf2 signaling pathway and its downstream effectors play a pivotal role in mitigating free radical-induced cellular damage. Emerging evidence suggests that ecdysteroids modulate the Nrf2 pathway, exemplified by β-ecdysterone’s neuroprotective effects through Nrf2 activation [44]. As a structural analog of β-ecdysterone, 25*R*-inokosterone belongs to the same phytosteroid class and exhibits potent ROS-scavenging activity, indicating inherent antioxidant properties. Mechanistically, 25*R*-inokosterone treatment significantly upregulated Nrf2 and Heme oxygenase 1 (HO-1) expression in SH-SY5Y cells, as confirmed by immunoblot analysis (Figure 12A). This molecular signature aligns with observed reductions in MPTP-induced apoptosis, evidenced by suppressed Bax expression and preserved MMP (Figure 11A,D). Critical validation was achieved through Nrf2 knockdown experiments, where the loss of Nrf2 abolished 25*R*-inokosterone’s protective effects against MPTP-induced neurotoxicity. Specifically, Nrf2 silencing reversed both HO-1 induction and Bax suppression (Figure 12B,C), while exacerbating mitochondrial dysfunction (Figure 12D)—thereby establishing Nrf2 activation as essential for the compound’s efficacy. As an upstream regulatory event independent of the core Nrf2 pathway, 25*R*-inokosterone downregulated MAOB and GSK-3β protein expression, which synergistically augmented the Nrf2-mediated neuroprotection against PD-like pathology (Figure 13). These multi-target actions position 25*R*-inokosterone as a promising therapeutic candidate for Parkinsonian disorders.

## 3. Discussion

AD and PD represent the two most prevalent neurodegenerative disorders globally. Epidemiological data reveal a staggering 30 million AD cases and over 6 million PD patients worldwide as of 2021 [45], with projections estimating >150 million neurodegenerative disease cases by 2050 [46]. Despite being the second most common neurodegenerative disorder, PD exhibits rapidly escalating disability-adjusted life years, imposing profound socioeconomic burdens through progressive motor impairment characterized by bradykinesia, rigidity, and debilitating tremors [47,48]. The hallmark pathology of PD involves selective degeneration of dopaminergic neurons in the substantia nigra pars compacta, accompanied by α-synuclein aggregates. Notably, chronic levodopa therapy—while initially effective—paradoxically exacerbates oxidative stress and accelerates mitochondrial dysfunction in dopaminergic neurons, contributing to treatment resistance [49,50]. This underscores an urgent need for disease-modifying therapeutics targeting upstream pathogenic mechanisms.

Environmental neurotoxicants critically influence PD etiopathogenesis. Rotenone, a widely used agricultural pesticide, induces Parkinsonism through selective inhibition of mitochondrial complex I, triggering dopamine-dependent oxidative stress and subsequent nigral neuron degeneration [51]. Similarly, MPTP—a classic rodent modeling agent—undergoes MAOB-mediated conversion to MPP^+^, which accumulates in dopaminergic neurons to provoke ROS overproduction and GSK-3β-mediated apoptotic cascades [52,53]. These convergent pathways highlight MAOB and GSK-3β as pivotal targets in the treatment of PD.

While synthetic MAOB inhibitors (e.g., safinamide) and GSK-3β inhibitors (e.g., indirubin-3′) demonstrate clinical efficacy, limitations such as a narrow therapeutic window and off-target effects remain significant challenges for these drugs. Natural products offer promising alternatives, yet the discovery of dual-target neuroprotective compounds remains largely unexplored. Through integrated computational-experimental approaches, we identified 25*R*-inokosterone as a novel neuroprotective agent whose core effect depends on Nrf2 activation, accompanied by modulation of MAOB/GSK-3β expression. Our strategy combined: (i) bioinformatic target prediction and pathway enrichment, (ii) microarray meta-analysis of PD patient/cell models, and (iii) structure-based molecular docking/pharmacophore modeling. Key findings included: (1) preferential binding affinity of ecdysterones to MAOB/GSK-3β active sites; (2) significant correlation between MAOB/GSK-3β expression levels and PD-related differentially expressed genes identified via microarray analysis; (3) superior neuroprotection effects of 25*R*-inokosterone against MPTP-induced cytotoxicity compared to those of β-ecdysterone.

Mechanistic insights revealed that 25*R*-inokosterone significantly attenuated MPTP metabolite-induced MMP loss in SH-SY5Y cells, with efficacy comparable to reference inhibitors (e.g., safinamide for MAOB or indirubin-3′ for GSK-3β). Immunofluorescence and Western blot analyses confirmed its downregulation of MAOB/GSK-3β protein expression, correlating with reduced levels of pro-apoptotic markers (Bax, cleaved caspase-3, and cleaved PARP). Crucially, the neuroprotective effect depended on Nrf2 pathway activation, as Nrf2 knockdown via siRNA markedly diminished protection. These findings propose a core-dependent regulatory framework: 25*R*-inokosterone exerts neuroprotection predominantly via Nrf2-mediated antioxidant and anti-apoptotic effects, with MAOB/GSK-3β expression downregulation acting as an upstream modulator of the ROS-apoptosis axis—a strategy that relies on the core Nrf2 pathway with synergistic upstream target modulation. Moreover, its natural origin suggests potential safety advantages over synthetic alternatives, though clinical validation remains necessary.

Limitations: There are several limitations in the present study that should be acknowledged.

First, there are some methodological limitations in the current experiments: (a) Undifferentiated SH-SY5Y cells were used as the in vitro model, which differ from primary dopaminergic neurons in endogenous MAOB expression levels and physiological phenotypes. Thus, the results obtained in this cell line cannot be directly extrapolated to in vivo physiological conditions. In addition, we evaluated the protein expression of MAOB but did not directly detect its enzymatic activity or the intracellular conversion of MPTP to MPP^+^. Therefore, the inference regarding MAOB-mediated neuroprotection is based on indirect evidence and remains preliminary. Further studies using primary neurons and direct enzymatic activity assays are warranted to validate the MAOB-targeted effect of 25*R*-inokosterone. (b) We demonstrated that 25*R*-inokosterone could downregulate the protein expression of GSK-3β and MAOB, which was supported by computational predictions and bioinformatics analysis. However, we have not performed direct in vitro enzymatic inhibition assays to confirm whether 25*R*-inokosterone can directly inhibit the enzymatic activity of these two enzymes. Future enzymatic inhibition assays with purified proteins are needed to verify the direct target inhibitory effect of 25*R*-inokosterone. (c) Our mechanistic experiments confirmed that the activation of the Nrf2/HO-1 pathway is the core mechanism of the neuroprotective effect of 25*R*-inokosterone, while the regulation of MAOB and GSK-3β may act as upstream modulators of this pathway. In the current study, we have not performed rescue experiments to verify the causal relationship between MAOB/GSK-3β regulation and Nrf2 activation. Further rescue experiments are needed to clarify the interaction between these pathways.

Second, in the preliminary cell assay, we used indirubin-3′ as the positive control for GSK-3β inhibition. Although indirubin-3′ has been widely used as a GSK-3β inhibitor in previous studies, it has relatively low selectivity over other kinases such as CDKs, which may limit the accuracy of the cell-based activity validation. In future studies, we will use more selective GSK-3β inhibitors such as CHIR99021 or SB216763, and even dual-target positive controls, to perform more in-depth cell-based experiments to verify the activity and advantages of 25*R*-inokosterone.

Third, we performed in silico ADME predictions to evaluate the pharmacokinetic properties of 25*R*-inokosterone. The results showed that 25*R*-inokosterone was predicted to have poor blood–brain barrier (BBB) permeability, which was consistent with the previous reports that ecdysteroids generally have limited CNS penetration due to their high polarity. This is mainly attributed to the multiple hydroxyl groups of 25*R*-inokosterone, which result in a high topological polar surface area, and it was also predicted to be a P-glycoprotein substrate, which would further limit its CNS penetration. This limitation suggests that the native 25*R*-inokosterone may not be suitable for direct use in the treatment of central nervous system diseases such as Parkinson’s disease. However, this does not negate the pharmacological activity of 25*R*-inokosterone against MAOB and GSK-3β. Future studies could focus on the structural optimization of 25*R*-inokosterone, such as prodrug modification or hydroxyl esterification, to improve its lipophilicity and BBB permeability, which would greatly expand its application prospects.

## 4. Materials and Methods

### 4.1. Materials

#### 4.1.1. Medicinal Plant

The decoction pieces of *A. bidentata* were purchased from Heng Gu Pharmaceutical Co., Ltd. (Bozhou, China) on 2 March 2023, and were identified by Associate Prof. Yaxin Zheng from Chengdu Medical College. A voucher specimen (No. ZJXUM-20230302-3) was deposited in the Department of Pharmacy, Jiaxing University.

#### 4.1.2. Regents and Experimental Assay Kits

The Dulbecco’s Modified Eagle Medium/Nutrient Mixture F-12 (DMEM/F12) cell culture medium, the Penicillin-Streptomycin solution, and the fetal bovine serum (FBS) were all obtained from Servicebio (Wuhan, China). The other materials, including the western and IP cell lysis buffer (catalog no. P0013), the phenylmethanesulfonyl fluoride (PMSF, catalog no. ST506), the BCA protein assay kit (catalog no. P0010S), the ECL Star kit (catalog no. P0018AS), the Triton X-100 (catalog no. P0096), the immunofluorescence staining kit with Alexa Fluor 555 (catalog no. P0179), the anti-fade mounting medium with DAPI (catalog no. P0131), the Annexin V-FITC Apoptosis Detection Kit (catalog no. C1062S), the MMP and Apoptosis Detection Kit (catalog no. C1071S), and the Lipo8000 transfection regent (catalog no. C0533), were all purchased from Beyotime Institute of Biotechnology Co., Ltd. (Shanghai, China). ELISA kits for dopamine, GSH, and LDH were purchased from the Meimian Institute of Biotechnology Co., Ltd. (catalog no. MM-1008H2, MM-0458H2, and MM-0354H2, Yancheng, China).

#### 4.1.3. Antibodies and siRNA

The primary antibodies and secondary antibody used were listed as follows: anti-GSK-3β (catalog no. bs-0023R, Bioss, Beijing, China), anti-MAOB (catalog no. bs-11297R, Bioss, China), anti-bax (catalog no. ab32503, Abcam, Cambridge, UK), anti-caspase-3 (catalog no. ab184787, Abcam, UK), anti-caspase-9 (catalog no. 9502, Cell Signaling Technology, Danvers, MA, USA), anti-PARP (catalog no. ab32138, Abcam, UK), anti-Nrf2 (catalog no. bs-1074R, Bioss, China; catalog no. ab62352, Abcam, UK), anti-HO1 (catalog no. ab52947, Abcam, UK; catalog no. p4115, Adamas, China), and horseradish peroxidase (HRP)-conjugated goat anti-rabbit IgG (catalog no. SA00001-2, Proteintech, Wuhan, China). The siRNA specific for Nrf2 was obtained from GenePharma (catalog no. 67156, Shanghai, China).

### 4.2. Natural Compounds Purification

#### 4.2.1. Methodologies

The HPLC preparation system, consisting of a Waters 2489 detector, a Waters 1525 pump, and a YMC-Pack ODS-A column (250 × 10 mm, 5 μm), was applied to separate the single compounds. MS data were measured on a Waters LCT Premier XE mass spectrometer (Milford, MA, USA). 1D and 2D NMR spectra were measured on a Bruker AM-400 NMR spectrometer (Ettlingen, Germany) with tetramethylsilane (TMS) as an internal standard. Silica gel (100–200 and 300–400 mesh, Qingdao Marine Chemical, Ltd., Qingdao, China) and Sephadex LH-20 (Pharmacia, Peapack, NJ, USA) were used for normal phase column chromatography, and TLC plates (GF254) were used for offering guidance on chemical isolation and examining the purity of single compounds.

#### 4.2.2. Extraction and Isolation

The segmented roots of *A. bidentata* (5 kg) were soaked in 95% EtOH overnight and refluxed three times (each for 2 h). The resulting solution was concentrated under vacuum at 60 °C to remove the EtOH, dispersed with deionized water, and progressively extracted with CH_2_Cl_2_ and EtOAc. The yields of CH_2_Cl_2_ (21.13 g) and EtOAc (15.5 g) extracts underwent silica gel column chromatography, eluted with petroleum ether:ethyl acetate (100:0 to 1:1) for the CH_2_Cl_2_ extract, and dichloromethane: methanol (100:0 to 1:1) for the EtOAc extract. This process resulted in a total of 8 fractions for the CH_2_Cl_2_ extract and 12 fractions for the EtOAc extract. For the CH_2_Cl_2_-derived fractions, fraction 4 (2.16 g, petroleum ether:ethyl acetate = 100:6–100:8) underwent another silica gel column separation using the petroleum ether-ethyl acetate solvent system for elution (100:0 to 100:100). Among the five obtained sub-fractions, sub-fraction 3 (0.96 g, petroleum ether:ethyl acetate = 100:8 to 100:15) and sub-fraction 4 (0.83 g, 100:20 to 100:50) underwent additional rounds of separation. For sub-fraction 3, it underwent Sephadex LH-20 separation (dichloromethane:methanol = 1:1) and HPLC preparation (MeOH:H_2_O = 65:35) sequentially, resulting in the isolation of compound **3** (3.7 mg). Sub-fraction 4, without Sephadex LH-20 separation, underwent HPLC preparation using the MeOH-H_2_O system (MeOH:H_2_O = 45:55). In this process, compound **1** (4.5 mg) and compound **2** (5 mg) were successfully prepared. Compound **8** (11.2 mg) was isolated from fraction 1 (1.59 g, petroleum ether: ethyl acetate = 100:0–100:1) using silica gel (39 mg, sub-fraction 2, petroleum ether: ethyl acetate = 100:8–100:20) and HPLC (MeOH: H2O = 65:35) sequentially. For the EtOAc-derived fractions, fractions 5 (3.42 g) and 6 (2.76 g) underwent simultaneous isolation using dichloromethane-methanol eluent systems at a gradient of 100:0–100:100. Sub-fraction 7 (1.06 g) from fraction 5, as well as sub-fractions 6 (0.94 g) and 10 (0.68 g) from fraction 6, were initially separated with a silica gel column using dichloromethane-methanol eluent systems. The indicated secondary fractions in each group were further purified using HPLC (MeOH-H_2_O system) at ratios of 37:63, 55:45, and 47:53, resulting in the isolation of compound **4** (5 mg) and compound **5** (3 mg) from sub-fraction 7, compound **6** (33 mg) from sub-fraction 6, and compound **7** (4.6 mg) from sub-fraction 10. Compounds **3**–**8** were identified as 5,6,7,2′-tetramethoxy-isoflavone, 25*S*-inokosterone, 25*R*-inokosterone, β-ecdysterone, stachysterone D, and bis(2-ethylhexyl)-phthalate. Compounds **1** and **2**, identified as novel isoflavonoids, were named achyraone A and achyraone B, respectively.

Notably, compounds **4** (25*S*-inokosterone) and 5 (25*R*-inokosterone) have only been isolated and reported from *Achyranthes bidentata* to date. For other known compounds, previous isolation from non-*Achyranthes* plant species has been well-documented: compound **3** (5,6,7,2′-tetramethoxyisoflavone) from *Anabasis brevifolia* [54]; compound **6** (β-ecdysterone) from *Rhaponticum carthamoides* [55] and *Klaseopsis chinensis* [56]; compound **7** (stachysterone D) from *Lychnis chalcedonica* [57]; and compound **8** (bis(2-ethylhexyl)-phthalate) from *Solanum lyratum* [18].

#### 4.2.3. Spectroscopic Data Analysis

**Figure A1:**
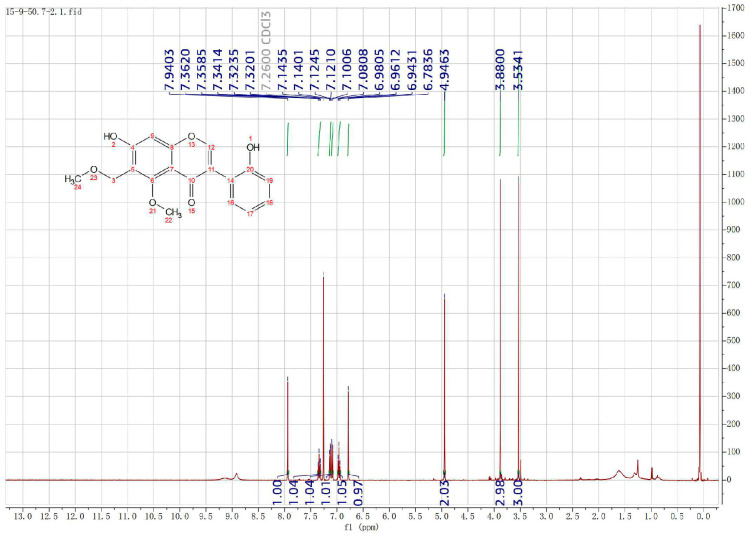
^1^H-NMR data (400 MHz) of compound **1** acquired using CDCl_3_ as solvent.

**Figure A2:**
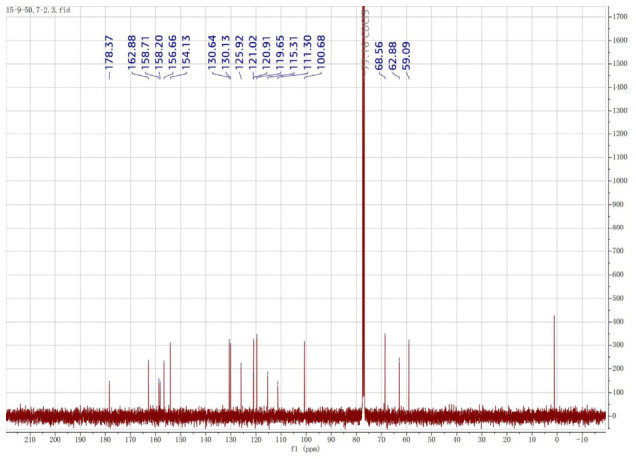
^13^C-NMR data (150 MHz) of compound **1** acquired using CDCl_3_ as solvent.

**Figure A3:**
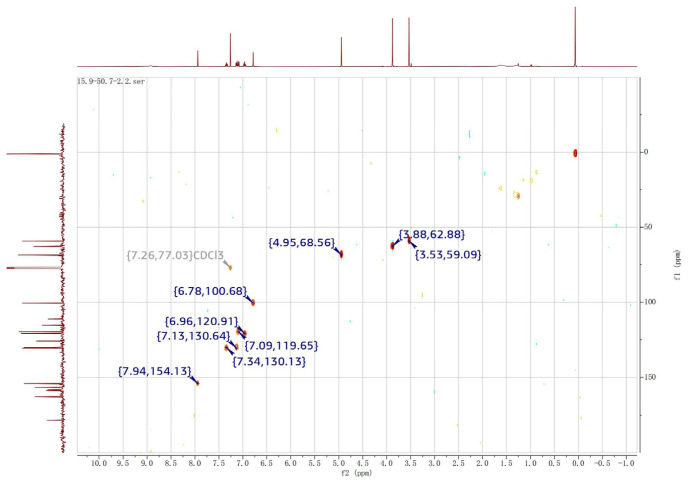
HSQC data (150 MHz) of compound **1** acquired using CDCl_3_ as solvent.

**Figure A4:**
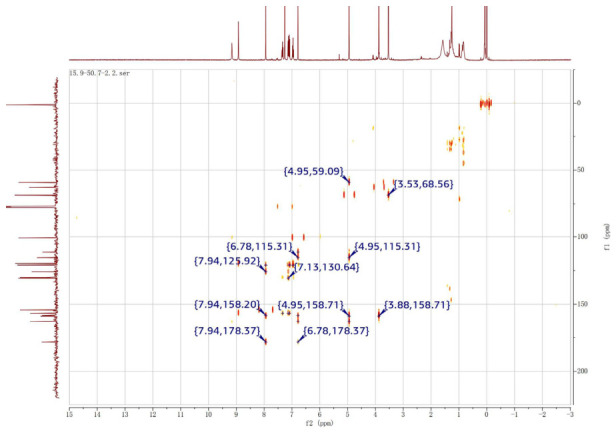
HMBC data (150 MHz) of compound **1** acquired using CDCl_3_ as solvent.

**Achyraone A** (1), light yellow amorphous powder; ^1^H and ^13^C-NMR data see Table 1 and Table 2, and Figure A1, Figure A2, Figure A3 and Figure A4 (+) TOF-MS *m*/*z* 351.0849 [M + Na]^+^, calcd for C_18_H_16_O_6_Na, 351.0845 (Figure A8).

**Figure A5:**
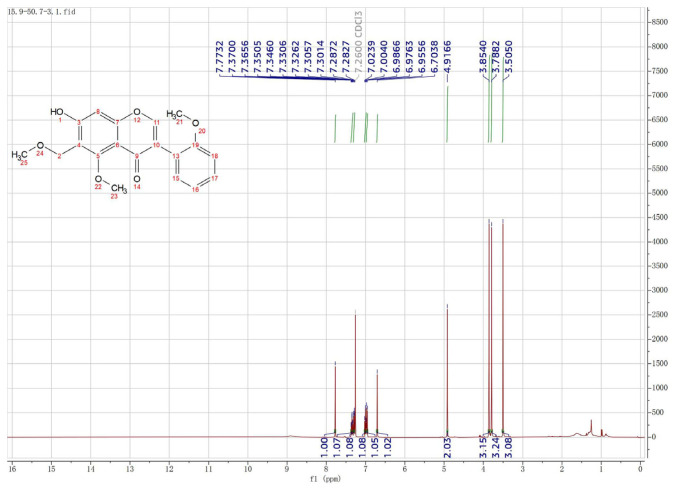
^1^H-NMR data (400 MHz) of compound **2** acquired using CDCl_3_ as solvent.

**Figure A6:**
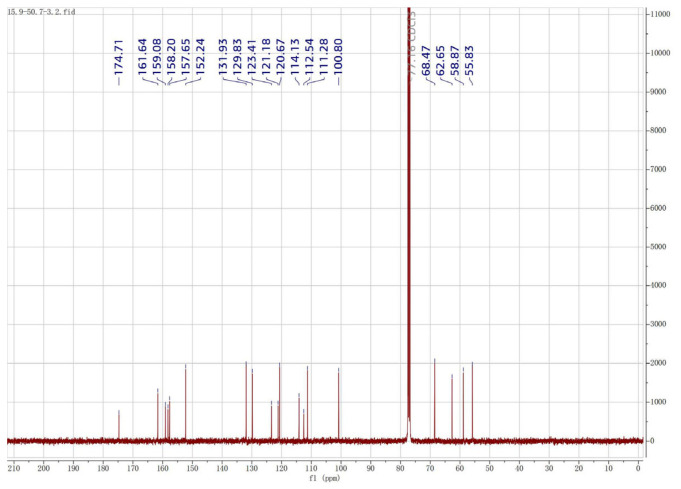
^13^C-NMR data (150 MHz) of compound **2** acquired using CDCl_3_ as solvent.

**Figure A7:**
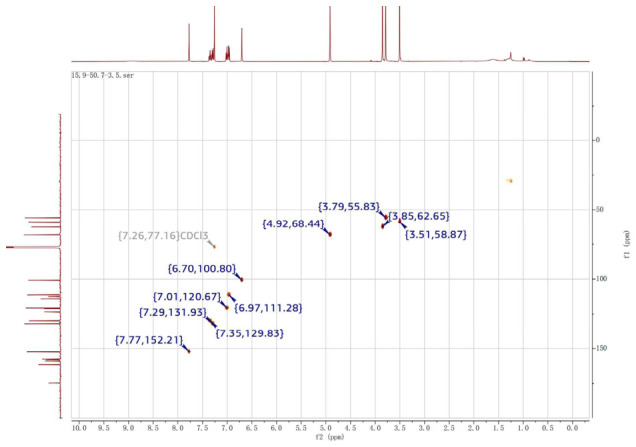
HSQC data (150 MHz) of compound **2** acquired using CDCl_3_ as solvent.

**Figure A8:**
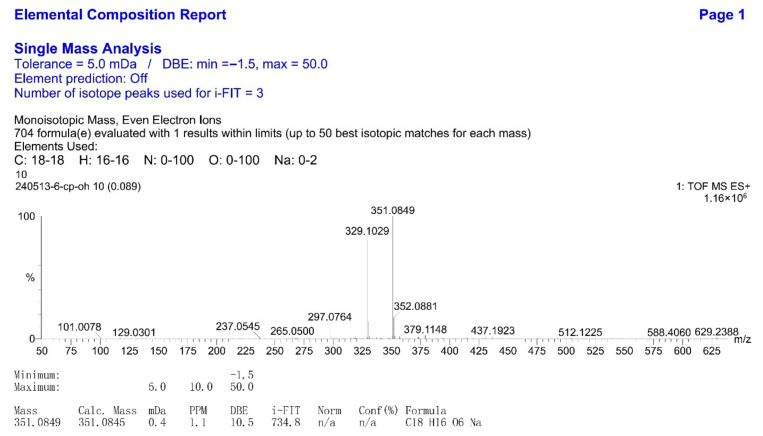
The HR-MS data of compound **1** under ES^+^ conditions.

**Figure A9:**
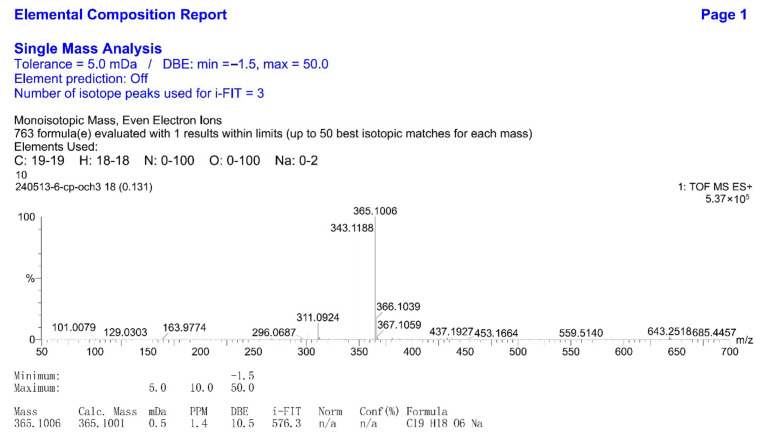
The HR-MS data of compound **2** under ES^+^ conditions.

**Achyraone B** (2), light yellow needles; ^1^H and ^13^C-NMR data see Table 1 and Table 2, and Figure A5, Figure A6 and Figure A7 (+) TOF-MS *m*/*z* 365.1006 [M + Na]^+^, calcd for C_19_H_18_O_6_Na, 365.1001 (Figure A9).

### 4.3. Bioinformatics and In Silico Analysis

#### 4.3.1. Screening of Key Compounds Targets on Neurodegenerative Disorder

We collected potential binding targets for our isolated compounds as well as for some reported *A. bidentata* compounds (belonging to the same kinds or analogs as isolated in this study) by using the Swiss Target Prediction online tool and preserved the ‘Homo sapiens’ labeled ones. Targets of compounds with a threshold value larger than 0.05 for the ‘Probability’ were kept. All these preliminarily screened targets corresponding to the compounds were then analyzed for functions and used to build score-based clusters in DAVID. The resulting targets (with an enrichment score larger than 5) recorded in terms of GO-BP, GO-MF, GO-CC, and KEGG pathways that are involved in PD were preserved.

#### 4.3.2. Gene Microarray Data

We collected both the whole substantia nigra (GSE20292) and the SH-SY5Y cell (GSE151808) chips from the GEO database. All chips were filtered for differentially expressed genes using the GENE-E analyzing tool. The filtered genes were then annotated for GO-BP, GO-MF, GO-CC, and the belonging signaling pathways with DAVID. Neurodegeneration-related records, especially for PD, were extracted and combined with the PD-related compound targets to construct PPI networks using STRING. Finally, both the PD-related compound targets and the filtered PD-related nodes in chips that are commonly embedded in the STRING annotations were assigned.

#### 4.3.3. Computational Simulation of Interactions Between Key Nodes and Compounds

After identifying GSK-3β and MAOB as core nodes interacting with neuroprotective compounds, the crystal structures of these proteins (PDB entries 7B6F for GSK-3β and 6FW0 for MAOB) were retrieved from the Protein Data Bank. Initially, molecular docking was performed to screen candidate compounds from *A. bidentata*, using binding modes, orientations, and energies as criteria compared with known expression modulators. This was followed by pharmacophore modeling to filter compounds based on fitness scores and spatial alignment with reference ligands. Finally, top-ranked molecules exhibiting optimal pharmacophore compatibility underwent in vitro anti-neurodegenerative evaluations.

#### 4.3.4. Validation of the Reliability of the Pharmacophore Models

To validate the pharmacophore models, we constructed a rigorous validation dataset. First, we retrieved highly active inhibitors of MAOB and GSK-3β from the ChEMBL database, with a screening threshold of IC_50_ < 100 nM, which served as the positive ligand sets for the validation of pharmacophores P10 (MAOB) and P5 (GSK-3β), respectively. After removing duplicate structures, excluding inorganic salts and fragmented small molecules, and performing energy minimization on the remaining molecules, we finally obtained 1352 positive active molecules for MAOB and 1389 positive active molecules for GSK-3β.

Due to the inaccessibility of the DUD-E database, we constructed target-specific negative decoy libraries following the standard construction protocol of DUD-E pharmacophore screening datasets. For MAOB, we integrated the available decoy molecules from the ZINC dataset; for GSK-3β, we developed an in-house Python 3.9 script to screen matching decoy molecules from the ZINC database. The constructed decoy molecules were strictly matched with the positive ligands of the corresponding targets in terms of key physicochemical properties, including molecular weight, logP, number of hydrogen bond donors, and number of hydrogen bond acceptors, ensuring that the decoys and active molecules shared highly similar physicochemical characteristics. Meanwhile, during the decoy library construction, we completely removed all known active molecules of the targets, guaranteeing that the decoy molecules had no target binding activity or pharmacological inhibitory effects.

Finally, we constructed 2080 target-specific negative decoys for MAOB and 2162 target-specific negative decoys for GSK-3β. The positive ligands and negative decoys were merged and imported into the Discovery Studio 3.5 software, where Principal classification labels were added to the molecular sets of the two targets (label 1 for decoy molecules, label 2 for active molecules). After pharmacophore molecular matching and mapping, we systematically calculated key validation metrics including ROC-AUC, enrichment factors EF 1%/5%/10%, and BEDROC early enrichment efficiency, to comprehensively evaluate the screening performance of the pharmacophore models.

#### 4.3.5. Validation of the Stability of the Receptor-Ligand Complexes

Molecular dynamics simulations were performed to validate the stability of the receptor-ligand complexes obtained from molecular docking. The simulations were carried out using the GROMACS 2022.2 software package. The Amber 14SB force field was applied to parameterize the protein residues, while the ligand was parameterized using the Antechamber program with the AM1-BCC charge model.

The complex systems were solvated in a cubic box of TIP3P water molecules, with a buffer distance of 1.0 nm between the protein surface and the box edge. To neutralize the system charge, Na^+^ and Cl^−^ were added to the system. Prior to the production simulation, energy minimization was performed using the steepest descent algorithm for 5000 steps, followed by the conjugate gradient algorithm for another 5000 steps, to remove steric clashes.

Subsequently, two-step equilibration simulations were performed. First, a 200 ps NVT equilibration was conducted at 298 K to stabilize the temperature of the system, with the protein and ligand backbone atoms restrained with a force constant of 10 kcal·mol^−1^·Å^−2^. Then, a 200 ps NPT equilibration was performed at 1 bar to stabilize the pressure of the system, with the same positional restraints.

After equilibration, we performed 100 ns production MD simulations for the complexes, without any positional restraints. The temperature was maintained at 300 K using the V-rescale thermostat, and the pressure was maintained at 1 bar using the Parrinello-Rahman barostat. The LINCS algorithm was applied to constrain the bonds involving hydrogen atoms, allowing a time step of 2 fs. The trajectories were saved every 10 ps for subsequent analysis.

The RMSD of the protein backbone atoms and the ligand heavy atoms was calculated using the gmx rmsd module in GROMACS to evaluate the dynamic stability of the systems throughout the simulations.

### 4.4. General Experimental Contents

#### 4.4.1. Cell Culture

The human neuroblastoma cell line SH-SY5Y was purchased from the American Type Culture Collection (ATCC, Manassas, VA, USA) and cultured in DMEM/F12 with 15% fetal bovine serum (FBS), 2 mM L-glutamine, 100 units/mL penicillin, and 0.1 mg/mL streptomycin. Sterile flasks containing cells and culture medium were incubated in a humidified incubator at 37 °C with CO_2_ supplementation to maintain the concentration at 5%.

#### 4.4.2. Cell Viability Assay

Initially, SH-SY5Y cells were divided into different groups, including a control group, a model group (MPTP group), positive control groups, MPTP + positive control treatment groups, compounds treatment groups, and MPTP + compounds treatment groups. Subsequently, cells at a density of 1 × 10^5^ cells/mL in the medium were seeded into a 96-well plate (each well containing 100 μL) and incubated in an incubator at 37 °C for 24 h. Following this, corresponding drugs were added 2 h before the MPTP treatment to cells in MPTP + positive control treatment groups and MPTP + compounds treatment groups. Cells in positive control groups and compound treatment groups received treatment with the corresponding drugs only, or without MPTP treatment. After further incubation for 24 h, 10 μL of CCK-8 was added to each well and incubated for 4 h. Absorbance for each well was measured at 450 nm using a microplate reader (Molecular Devices, Gemini EM, San Jose, CA, USA), and the viabilities for each group were calculated.

To establish the MPTP-induced SH-SY5Y cell lesion model, a range of MPTP concentrations was applied to cells, and cell viability was assessed using the CCK-8 assay. The criteria for a successful MPTP-induced SH-SY5Y cell lesion model in this study included a significant cytoplasmic vacuolation and a noticeable decrease in cell viability (ranging from 45% to 65%). The concentration that met these criteria was deemed appropriate for further use in subsequent experiments.

#### 4.4.3. ELISA

To quantify the dopamine content, GSH level in the modeled SH-SY5Y cells, and the LDH standard in the cell culture medium, we conducted three specific ELISA assays. Initially, cells were seeded into T25 cell culture flasks and randomly assigned to control, model, positive control, and compound treatment groups. Each flask was filled with 7.8 mL DMEM/F12 (containing 7.8 × 10^5^ cells). After treatment with MPTP or the corresponding drugs for the indicated time, cells designated for dopamine and GSH assays were rinsed with PBS and collected into separate Eppendorf tubes. Following cell counting, lysis, and centrifugation, the supernatant from each group was collected, and dopamine and GSH contents were measured using ‘MM-1008H2’ and ‘MM-0458H2’ following the manufacturer’s instructions. As for the LDH assay, it involved collecting the cell culture media. After a 5 min centrifugation at 1000× *g*, the supernatant from each group was collected, and the LDH standard was measured using ‘MM-0354H2’.

#### 4.4.4. Transfection of siRNA

In this phase, we utilized Lipo8000 as a transfection reagent to transfer si-Nrf2 into SH-SY5Y cells, with si-NC serving as the transfection control. During the siRNA transfection process, cells were initially seeded into a 6-well plate and cultured for 24 h. Once reaching a cell confluence of approximately 70%, the culture medium was replaced with 2 mL of freshly prepared medium. On another front, 125 μL of penicillin-streptomycin-FBS-free DMEM/F12 was added to each individual Eppendorf tube, along with 100 pmol of si-Nrf2 or si-NC, and 4 μL of Lipo8000, sequentially supplemented. After gentle mixing and a 20 min incubation at room temperature, 125 μL of the assembled siRNA-Lipo8000 liposomes were ultimately added to the corresponding wells for siRNA transfection. Following a 6 h incubation of the culture plate at 37 °C, the established Nrf2-knockdown SH-SY5Y cells will be employed in the subsequent experiments as needed.

#### 4.4.5. Immunoblotting Assay

For the immunoblotting experiments, all processed cells were initially rinsed with ice-cold PBS and scraped down with a cell scraper. Cells in each group were then dispersed in new ice-cold PBS and centrifuged at 1000× *g* to obtain cell pellets. The ice-cold western and IP cell lysis buffer, containing 1 mM PMSF, was added to the cell pellets (100 μL lysis buffer for 1 × 10^6^ cells) and lysed on ice for 50 min. The supernatant of all cell lysates was collected after centrifugation at 12,000 rpm, and the total proteins in the cell lysates were quantified using the BCA protein concentration method. Primary antibodies for GSK-3β, MAOB, Nrf2, and PARP were diluted at 1:1000 with 5% skimmed milk (dissolved in TBST), while HO-1 and caspase-9 were diluted at 1:2000, and β-actin at 1:5000. After transferring proteins onto PVDF membranes, they were blocked with 5% skimmed milk at 25 °C for 2 h and incubated with corresponding primary antibodies at 4 °C overnight. All membranes were then washed with TBST (3 times, each for 5 min) and further incubated with the HRP-conjugated secondary antibody (diluted at 1:5000 with 1% skimmed milk). After another round of washing with TBST (3 times, each for 5 min), signals for each protein were developed using the Beyotime ECL Star kit and observed with an Amersham Image Quant 800 chemiluminescence imaging analysis system (Cytiva, Marlborough, MA, USA).

#### 4.4.6. Immunofluorescence Assay

The immunofluorescence assay was employed to detect the expression of GSK-3β, MAOB and cleaved caspase-3 in different groups of SH-SY5Y cells. The detailed experiment proceeded as follows: cells were cultured in T25 cell culture flasks until reaching 80% confluence. Subsequently, they were washed twice with sterile-filtered PBS and digested with trypsin to release cell adhesion. The cells were diluted with PBS and centrifuged at 1000× *g* for 5 min. The resulting cell pellet was resuspended in DMEM/F12 medium and dropped onto a cover glass in a 6-well cell culture plate, then incubated for 24 h at 37 °C in an incubator. The prepared cell climbing sheets were then treated with the corresponding concentrations of compounds or MPTP for the required time. Once acquired, all the cell climbing sheets were treated with 4% paraformaldehyde (4% PFA) overnight at 4 °C. The following day, they were rinsed with PBS and perforated with TBSTx (final concentration of 0.1% Triton X-100 in TBS solution) for the attached cells. Subsequent procedures, including blocking with 5% BSA and incubation with primary/fluorescein-bonded secondary antibodies, were conducted similarly to the immunoblotting assay. The processed cell climbing sheets were finally sealed with neutral balsam on slides, and the expressions of GSK-3β, MAOB and cleaved caspase-3 in SH-SY5Y cells were observed using a laser confocal microscope (FV3000, Olympus, Tokyo, Japan).

### 4.5. Cell Morphology

#### 4.5.1. TUNEL Staining

In this section, we employed TUNEL staining to assess the quantity of apoptotic cells, both with and without drug treatment. Initially, cells dispersed in 100 μL DMEM/F12 were seeded in a 96-well plate at a density of 1 × 10^5^ cells/mL. The cells were then categorized into several groups, including a control group, a model group, positive control groups, and groups treated with compounds. Following the MPTP or drug treatment of cells, conducted as described in the cell viability assay, they underwent a round of PBS rinsing. The subsequent treatment procedures for each group of cells, comprising a 30 min fixation with 4% PFA, another round of PBS rinsing, a 5 min permeabilization with TBSTx at room temperature, another round of PBS rinsing, a 60 min incubation with the TUNEL testing solution at 37 °C, and a final three rounds of PBS rinsing, were carried out sequentially. For result detection, 100 μL of Antifade Mounting Medium with DAPI was added to cells in each well, and the fluorescence intensity was observed using an Olympus CKX53 fluorescence microscope (Olympus, Tokyo, Japan).

#### 4.5.2. Mito-Tracker Red CMXRos Staining

The Mito-Tracker Red CMXRos staining assay conducted in this section aimed to reveal compound-mediated neuroprotection, based on the MMP status. Cells were initially seeded in a 12-well plate containing 1.4 mL DMEM/F12 in each well (1.4 × 10^5^ cells per well). Following the same grouping procedure as described in the ‘TUNEL staining’ and a 24 h incubation in an incubator at 37 °C, cells in each group were treated with MPTP and corresponding drugs, as outlined in the ‘Cell viability assay’. After completing PBS rinsing, staining, a 30 min incubation in the dark, a new round of PBS rinsing, and the addition of DMEM/F12 in that order, the experimental results were immediately observed, and high-resolution pictures were captured using the Olympus CKX53.

#### 4.5.3. DCFH-DA Staining

A DCFH-DA-based ROS visualization assay was conducted in PD-model cells as another reflection of cellular mitochondrial function. For this, cells preincubated in a 6-well culture plate (3 mL, at a density of 1 × 10^5^ cells/mL, for 24 h) underwent PBS rinsing, DCFH-DA working fluid incubation (10 μmol/L DCFH-DA in serum-free DMEM/F12), and three rounds of serum-free DMEM/F12 rinsing in sequence. Consistent with the Mito-Tracker Red CMXRos assay, the Olympus CKX53 microscope was also utilized to detect and record the experimental results.

#### 4.5.4. Rhodamine 123 Staining

To validate the MMP observations from MitoTracker Red CMXRos staining and evaluate the impact of siRNA transfection on 25*R*-inokosterone-mediated neuroprotection, an independent assay was performed using Rhodamine 123 fluorescent dye following the manufacturer’s instructions. Briefly, cells were seeded in 24-well plates at a density of 3 × 10^4^ cells/well in 1 mL complete medium and incubated for 24 h to achieve 50~70% confluence. Cells in corresponding groups were then transfected with either si-NC or si-Nrf2 using appropriate transfection reagents. After 12 h, the transfection mixture was replaced with fresh complete medium. At 72 h post-transfection, cells underwent secondary treatments (identical to those described in “Mito-Tracker Red CMXRos staining” except for dosage adjustments) and were further cultured for 24 h. Following treatment, cells were gently washed three times with PBS and incubated with Rhodamine 123 working solution (prepared by diluting the 1000× stock 1:1000 in diluent) at 37 °C for 30 min in the dark. The staining reaction was terminated by replacing the working solution with complete medium after two rounds of washes. Finally, images were captured immediately using an Olympus CKX53 fluorescence microscope.

### 4.6. Flow Cytometry

#### Annexin V-FITC/PI Dual Staining

Taking as supplementary evidence the observed TUNEL staining result, we continued to use Annexin-V/PI dual staining to have a deeper understanding of the refined compounds mediated neuroprotection. Experimental groups in this part were divided as mentioned in the TUNEL staining assay. Procedures for the Annexin-V/PI dual staining assay of the flow cytometry was listed as follows: seed cells in 6-well plate (each well for 2.5 × 10^5^ at 2.5 mL DMEM/F12); treat with MPTP or indicated drugs at corresponding time point; collect culture mediums and the cell washing exhausted PBS; digest cells with trypsin within indicated time and disperse with the collected liquid (mixture of culture mediums and PBS); centrifuge at 1000× *g* and collect cells; dilute cells with Annexin V-FITC binding solution; add Annexin V-FITC and propidium iodide (PI) successively; incubate in dark environment for 15 min; observe experimental result with flow cytometry.

### 4.7. Statistical Analysis

All quantitative data were presented as mean ±SD. Biological replicates were set as three independent experiments (n = 3), and technical replicates were performed in triplicate wells for each sample in all assays including CCK-8, ELISA, and Western blot. For the multi-concentration and multi-time-point CCK-8 cell viability assay, as well as other intergroup comparisons, one-way analysis of variance (ANOVA) was performed using SPSS 16.0 software, followed by Tukey’s multiple comparisons test for pairwise post hoc analysis. A *p*-value < 0.05 was considered statistically significant.

## 5. Conclusions

This study systematically evaluated the anti-PD activity of eight compounds isolated from *A. bidentata* through integrated bioinformatic prediction, network pharmacology analysis, in vitro activity screening, and molecular mechanism investigation. Key findings include: (1) ecdysteroids from *A. bidentata* generally exhibit anti-PD potential, with 25*R*-inokosterone exerting core neuroprotective efficacy via Nrf2/HO-1 activation, with prominent upstream modulatory effects on MAOB and GSK-3β protein expression; (2) in MPTP-induced SH-SY5Y cell lesion models, 25*R*-inokosterone (0.78 μM) significantly reversed MMP loss; (3) pharmacological characterization revealed that 25*R*-inokosterone exhibited no significant cytotoxicity at effective concentrations, outperforming the reference expression inhibitors safinamide and indirubin-3’. These findings not only highlight the unique advantages of 25*R*-inokosterone as a multi-target natural product but also establish it as a promising lead compound for developing next-generation anti-PD therapeutics capable of both symptom management and disease modification.

## Figures and Tables

**Table 1:** The ^1^H-NMR spectra data of compounds **1**–**8**.

Proton	1	2	3	Proton	4	5	6	7	**Proton**	**8**
	*δ* in ppm *J* in Hz				*δ* in ppm *J* in Hz					***δ* in ppm** ***J* in Hz**
**1**	-	-	-	**1**	2.16 (1H, m) 1.94 (1H, m)	2.16 (1H, m) 1.94 (1H, m)	1.43 (1H, m) 1.78 (1H, m)	1.43 (1H, t, 12.6) 1.78 (1H, m)	**1**	-
**2**	7.94 (1H, s)	7.77 (1H, s)	7.79 (1H, s)	**2**	4.19 (1H, m)	4.19 (1H, m)	3.83 (1H, dt, 3.9, 11.6)	3.84 (1H, dt, 3.9, 11.7)	**2**	-
**3**	-	-	-	**3**	4.25 (1H, brs)	4.26 (1H, brs)	3.95 (1H, d, 3.0)	3.95 (1H, d, 3.3)	**3**	7.70 (1H, m)
**4**	-	-	-	**4**	2.03 (1H, m) 1.82 (1H, m)	2.05 (1H, m) 1.82 (1H, m)	1.67 (1H, m) 1.75 (1H, m)	1.70 (1H, m) 1.75 (1H, m)	**4**	7.52 (1H, m)
**5**	-	-	-	**5**	3.03 (1H, dd, 3.7, 13.2)	3.03 (1H, dd, 3.8, 13.1)	2.38 (1H, m)	2.37 (1H, m)	**5**	7.52 (1H, m)
**6**	-	-	-	**6**	-	-	-	-	**6**	7.70 (1H, m)
**7**	-	-	-	**7**	6.27 (1H, d, 2.5)	6.28 (1H, d, 2.5)	5.81 (1H, d, 2.5)	5.81 (1H, d, 2.5)	**7**	-
**8**	6.78 (1H, s)	6.70 (1H, s)	6.69 (1H, s)	**8**	-	-	-	-	**8**	-
**9**	-	-	-	**9**	3.61 (1H, m)	3.62 (1H, m)	3.15 (1H, m)	3.16 (1H, m)	**1’**	4.22 (2H, m)
**10**	-	-	-	**10**	-	-	-	-	**2’**	1.68 (1H, m)
**11**	4.95 (2H, s)	4.92 (2H, s)	-	**11**	1.75 (1H, m) 1.89 (1H, m)	1.75 (1H, m) 1.89 (1H, m)	1.68 (1H, m) 1.80 (1H, m)	1.69 (1H, m) 1.80 (1H, m)	**3′**	1.33 (2H, m)
**1′**	-	-	-	**12**	2.04 (1H, m) 2.63 (1H, td, 4.8, 12.9)	2.05 (1H, m) 2.64 (1H, td, 4.6, 12.9)	1.83 (1H, m) 2.14 (1H, td, 4.8, 13.0)	1.85 (1H, m) 2.15 (1H, td, 4.6, 13.0)	**4′**	1.30 (2H, m)
**2′**	-	-	-	**13**	-	-	-	-	**5′**	1.30 (2H, m)
**3′**	7.09 (1H, brd, 7.9)	6.97 (1H, brd, 8.3)	6.96 (1H, brd, 8.3)	**14**	-	-	-	-	**6′**	0.91 (3H, t, 7.5)
**4′**	7.34 (1H, td, 1.4, 7.6)	7.35 (1H, td, 1.8, 7.8)	7.35 (1H, td, 1.8, 7.7)	**15**	1.91 (1H, m) 2.17 (1H, m)	1.91 (1H, m) 2.17 (1H, m)	2.00 (1H, m) 1.57 (1H, m)	1.98 (1H, m) 1.60 (1H, m)	**1″**	4.22 (2H, m)
**5′**	6.96 (1H, t, 7.7)	7.01 (1H, t, 7.9)	7.00 (1H, td, 1.1, 7.4)	**16**	2.05 (1H, m) 2.48 (1H, m)	2.07 (1H, m) 2.50 (1H, m)	1.95 (1H, m) 1.75 (1H, m)	1.95 (1H, m) 1.71 (1H, m)	**2″**	1.68 (1H, m)
**6′**	7.13 (1H, dd, 1.4, 7.6)	7.29 (1H, dd, 1.8, 7.4)	7.31 (1H, dd, 1.8, 7.5)	**17**	2.97 (1H, t, 9.2)	2.97 (1H, t, 9.1)	2.39 (1H, m)	2.37 (1H, m)	**3″**	1.33 (2H, m)
**5-OCH_3_**	3.88 (3H, s)	3.85 (3H, s)	3.94 (3H, s)	**18**	1.24 (3H, s)	1.25 (3H, s)	0.89 (3H, s)	0.84 (3H, s)	**4″**	1.30 (2H, m)
**6-OCH_3_**	-	-	3.91 (3H, s)	**19**	1.08 (3H, s)	1.09 (3H, s)	0.97 (3H, s)	0.97 (3H, s)	**5″**	1.30 (2H, m)
**7-OCH_3_**	-	-	3.95 (3H, s)	**20**	-	-	-	-	**6″**	0.91 (3H, t, 7.5)
**11-OCH_3_**	3.53 (3H, s)	3.51 (3H, s)	-	**21**	1.59 (3H, s)	1.61 (3H, s)	1.19 (3H, s)	1.21 (3H, s)	**2′-C_2_H_5_**	1.41 (2H, m) 0.89 (3H, t, 6.8)
**2′-OCH_3_**	-	3.79 (3H, s)	3.78 (3H, s)	**22**	3.87 (1H, brd, 10.2)	3.88 (1H, brd, 10.2)	3.33 (1H, m)	3.93 (1H, m)	**2″-C_2_H_5_**	1.41 (2H, m) 0.89 (3H, t, 6.8)
				**23**	1.63 (1H, m) 1.94 (1H, m)	1.70 (1H, m) 1.77 (1H, m)	1.30 (1H, m) 1.66 (1H, m)	1.76 (1H, m) 1.91 (1H, m)		
				**24**	1.43 (1H, m) 2.26 (1H, m)	1.78 (1H, m) 1.93 (1H, m)	1.75 (1H, m) 1.45 (1H, m)	1.75 (1H, m) 1.71 (1H, m)		
				**25**	1.82 (1H, m)	1.82 (1H, m)	-	-		
				**26**	3.68 (1H, d, 6.6) 3.78 (1H, dd, 5.4, 10.3)	3.68 (1H, d, 5.6) 3.74 (1H, m)	1.20 (3H, s)	1.25 (3H, s)		
				**27**	1.05 (3H, d, 6.6)	1.06 (3H, d, 6.2)	1.21 (3H, s)	1.24 (3H, s)		

**Table 2:** The ^13^C-NMR spectra data of compounds **1**–**8**.

Carbon	1	2	3	Carbon	4	5	6	7	Carbon	8
	*δ* in ppm				*δ* in ppm					*δ* in ppm
**1**	-	-	-	**1**	37.9	37.9	37.4	37.4	**1**	132.6
**2**	154.1	152.2	152.2	**2**	68.0	68.0	68.7	68.7	**2**	132.6
**3**	125.9	123.4	123.1	**3**	68.0	68.0	68.5	68.5	**3**	128.9
**4**	178.4	174.7	174.8	**4**	32.4	32.4	32.9	32.9	**4**	131.0
**5**	158.7	159.1	153.1	**5**	51.3	51.3	51.8	51.8	**5**	131.0
**6**	115.3	114.1	140.6	**6**	203.4	203.4	206.5	206.5	**6**	128.9
**7**	162.9	161.6	157.6	**7**	121.6	121.6	122.1	122.1	**7**	167.9
**8**	100.7	100.8	96.2	**8**	166.0	166.0	168.0	168.0	**8**	167.9
**9**	158.2	158.2	154.8	**9**	34.4	34.3	35.1	35.1	**1′**	68.3
**10**	111.3	112.5	113.9	**10**	38.6	38.6	39.3	39.3	**2′**	38.9
**11**	68.6	68.4	-	**11**	21.4	21.3	21.5	21.8	**3′**	30.5
**1′**	121.0	121.2	121.1	**12**	31.7	31.6	32.5	32.3	**4′**	29.1
**2′**	156.7	157.7	157.7	**13**	48.0	48.0	49.0	49.0	**5′**	23.1
**3′**	119.7	111.3	111.3	**14**	84.1	84.1	85.2	85.2	**6′**	14.2
**4′**	130.1	129.8	129.8	**15**	31.9	31.9	31.8	31.7	**1″**	68.3
**5′**	120.9	120.7	120.6	**16**	21.6	21.5	21.5	21.5	**2″**	38.9
**6′**	130.6	131.9	132.0	**17**	50.0	49.9	50.5	51.8	**3″**	30.5
**5-OCH_3_**	62.9	62.7	62.3	**18**	17.7	17.8	18.1	18.1	**4″**	29.1
**6-OCH_3_**	-	-	61.7	**19**	24.4	24.3	24.4	24.4	**5″**	23.1
**7-OCH_3_**	-	-	56.4	**20**	76.7	76.6	77.9	77.0	**6″**	14.2
**11-OCH_3_**	59.1	58.9	-	**21**	21.0	21.0	21.1	20.7	**2′-C_2_H_5_**	23.9 11.1
**2′-OCH_3_**	-	55.8	55.9	**22**	77.2	76.7	78.4	85.5	**2″-C_2_H_5_**	23.9 11.1
				**23**	30.1	29.8	27.3	28.5		
				**24**	31.9	31.6	42.4	39.6		
				**25**	36.7	36.3	71.3	81.8		
				**26**	67.2	68.0	29.7	28.4		
				**27**	17.8	16.9	29.0	29.0		

**Table 3:** Correspondence of key nodes and *Achyranthes bidentata* Bl. compounds about neurodegenerative disorders.

Class	Compounds	Terms	*p*-Values	Diseases	Key Nodes
**Ecdysterones**	Stachysterone D; β-ecdysterone; 25*R*-inokosterone	GO-MF: Tau-protein kinase activity	2.91 × 10^−3^	Alzheimer’s disease	GSK3B
	Stachysterone D; β-ecdysterone; 25*R*-inokosterone	KEGG-Pathway: Alzheimer′s disease	5.05 × 10^−6^	Alzheimer’s disease	GSK3B
	Stachysterone D; Achyranthesterone A; Niuxixinsterone A; Polypodine B				mTOR
	Achyranthesterone A; β-ecdysterone; Niuxixinsterone A; 25*S*-inokosterone; 20*R*, 22*R*-2β, 3β, 20, 22, 26-pentahydroxy-cholestan-7,12-dien-6-one				PSEN2
	20,22-*O*-*R*-ethylidene-20-hydroxyecdysone; 25*S*-inokosterone-20,22-acetonide; 20-hydroxyecdysone-20,22-monoacetonide; β-ecdysterone; 25*R*-inokosterone	KEGG-Pathway: Parkinson′s disease	7.38 × 10^−5^	Parkinson’s disease	MAOB
	20*R*, 22*R*-2β, 3β, 20, 22, 26-pentahydroxy-cholestan-7,12-dien-6-one; Achyranthesterone A; Polypodine B; β-ecdysterone; 25*R*-inokosterone; 25*S*-inokosterone				SLC6A3
	Stachysterone D; β-ecdysterone; 25*R*-inokosterone	KEGG-Pathway: Dopaminergic synapse	5.13 × 10^−7^	Parkinson’s disease	GSK3B
	20,22-*O*-*R*-ethylidene-20-hydroxyecdysone; 25*S*-inokosterone-20,22-acetonide; 20-hydroxyecdysone-20,22-monoacetonide; β-ecdysterone; 25*R*-inokosterone				MAOB
	20*R*, 22*R*-2β, 3β, 20, 22, 26-pentahydroxy-cholestan-7,12-dien-6-one; Achyranthesterone A; Polypodine B; β-ecdysterone; 25*R*-inokosterone; 25*S*-inokosterone				SLC6A3
	Niuxixinsterone B; Achyranthesterone A; 20*R*, 22*R*-2β, 3β, 20, 22, 26-pentahydroxy-cholestan-7,12-dien-6-one; β-ecdysterone; 25*R*-inokosterone; 25*S*-inokosterone; Polypodine B	GO-MF: Dopamine binding	3.50 × 10^−3^	Parkinson’s disease	DRD1
	20*R*, 22*R*-2β, 3β, 20, 22, 26-pentahydroxy-cholestan-7,12-dien-6-one; Achyranthesterone A; Polypodine B; β-ecdysterone; 25*R*-inokosterone; 25*S*-inokosterone				SLC6A3
	20*R*, 22*R*-2β, 3β, 20, 22, 26-pentahydroxy-cholestan-7,12-dien-6-one; Stachysterone D	GO-BP: synaptic transmission, dopaminergic	4.15 × 10^−4^	Parkinson’s disease	CDK5
	Niuxixinsterone B; Achyranthesterone A; 20*R*, 22*R*-2β, 3β, 20, 22, 26-pentahydroxy-cholestan-7,12-dien-6-one; β-ecdysterone; 25*R*-inokosterone; 25*S*-inokosterone; Polypodine B				DRD1
	Niuxixinsterone A; Niuxixinsterone B; 20*R*, 22*R*-2β, 3β, 20, 22, 26-pentahydroxy-cholestan-7,12-dien-6-one; β-ecdysterone; 25*R*-inokosterone; 25*S*-inokosterone				DRD2
	Niuxixinsterone B; Achyranthesterone A; 20*R*, 22*R*-2β, 3β, 20, 22, 26-pentahydroxy-cholestan-7,12-dien-6-one; β-ecdysterone; 25*R*-inokosterone; 25*S*-inokosterone; Polypodine B				DRD3
**Polyphenol derivatives**	Wogonin; Astragalin; Caffeic acid; Isoquercetin; Rutin	KEGG-Pathway: Alzheimer′s disease	1.58 × 10^−6^	Alzheimer’s disease	GSK3B
	Achyraone A; Achyraone B				mTOR
	Achyraone A				GRIN2B
	Wogonin; Caffeic acid; Astragalin; Isoquercetin; Rutin				APP
	Wogonin; Astragalin; Caffeic acid; Isoquercetin; Rutin	KEGG-Pathway: Dopaminergic synapse	2.20 × 10^−6^	Parkinson’s disease	GSK3B
	Achyraone B;5,6,7, 2’-tetramethoxy-isoflavone; Caffeic acid				MAOB
	Achyraone B; 5,6,7, 2’-tetramethoxy-isoflavone; Caffeic acid	GO-BP: dopamine catabolic process	1.97 × 10^−2^	Parkinson’s disease	MAOB
	Astragalin				COMT

Note: Monoamine oxidase B (MAOB), Glycogen synthase kinase-3β (GSK-3β).

**Table 4:** Binding modes and binding energies of positive ligands and top 5 dock-scored *Achyranthes bidentata* Bl. compounds with GSK-3β and MAOB.

MAOB		GSK-3β	
Binding mode	-CDOCKER interaction energy	Binding mode	-CDOCKER interaction energy
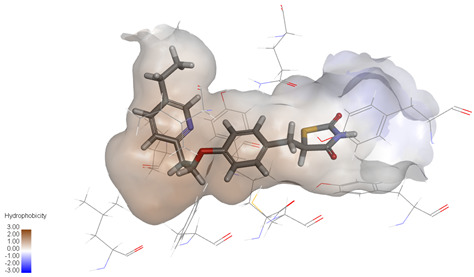 Pioglitazone (positive control)	52.5142	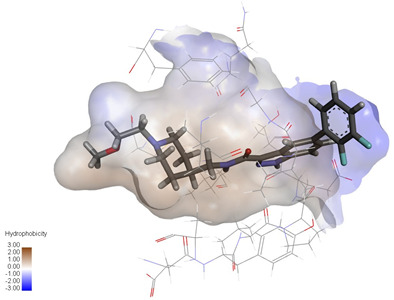 PDB: 6TCU (positive control)	49.5199
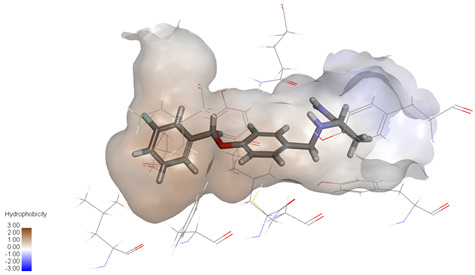 Safinamide (positive control)	51.6137	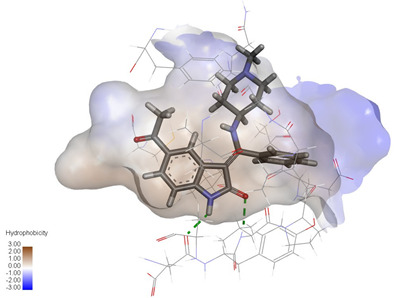 BI-91BS (positive control)	47.1882
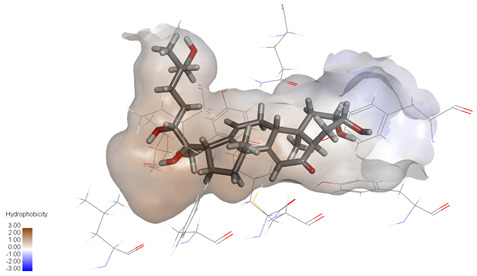 (20*R*,22*R*)-2β,3β,20,22,26-pentahydroxy-cholestan-7,12-dien-6-one	43.78239	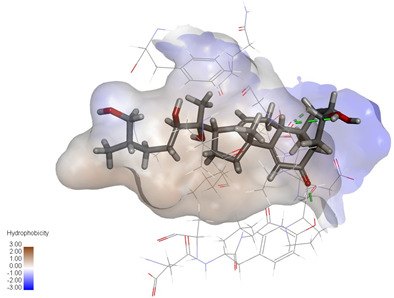 (20*R*,22*R*)-2β,3β,20,22,26-pentahydroxy-cholestan-7,12-dien-6-one	55.0985
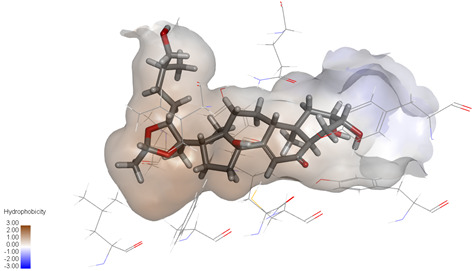 (25*S*)-20,22-O-(*R*-ethylidene)-inokosterone	33.9403	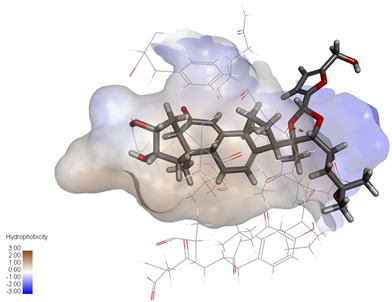 Niuxixinsterone B	54.9859
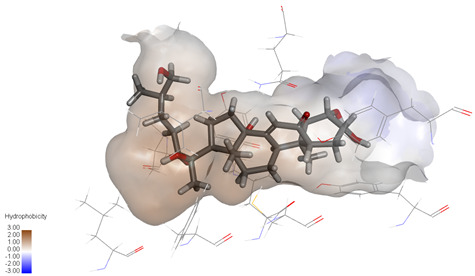 (25*R*)-inokosterone	28.2977	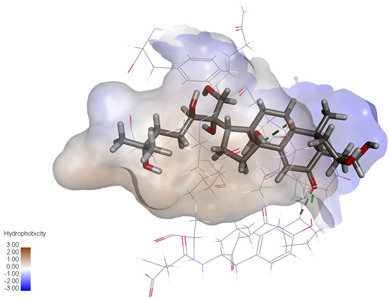 Achyranthesterone A	54.4297
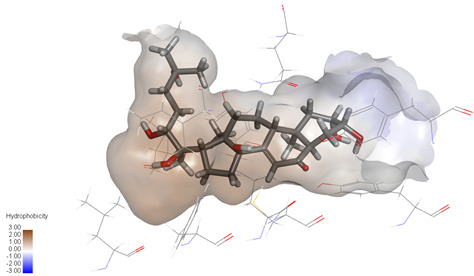 β-ecdysterone	26.2112	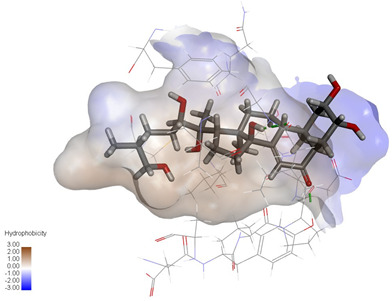 (25*R*)-inokosterone	53.4706
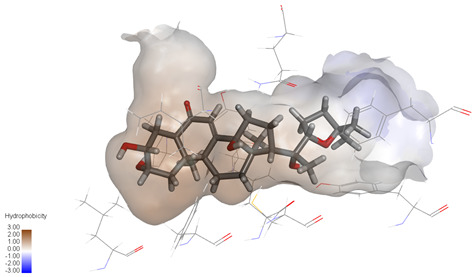 Stachysterone D	13.7666	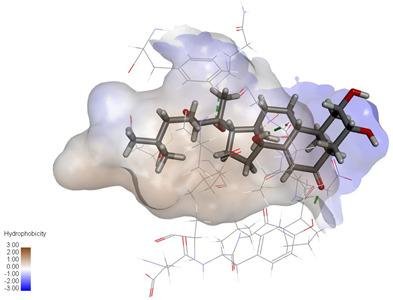 β-ecdysterone	47.4114

**Table 5:** The common feature-based pharmacophore generation fit for GSK-3β and MAOB.

GSK-3β				MAOB			
No.	Features	Direct Hit	Partial Hit	No.	Features	Direct Hit	Partial Hit
1	HDAA	10	0	1	HDAA	3	0
2	HDAA	10	0	2	HDAA	3	0
3	HDAA	10	0	3	HHDA	3	0
4	HDAA	10	0	4	HHDA	3	0
5	HDAA	10	0	5	RHA	3	0
6	HDAA	10	0	6	RHA	3	0
7	HDAA	10	0	7	RHA	3	0
8	HAA	10	0	8	RHA	3	0
9	HAA	10	0	9	RHA	3	0
10	DAA	10	0	10	HHDA	3	0

**Table 6:** Evaluation of all isolated *A*. *bidentata* compounds against 1-Methyl-4-phenyl-1,2,3,6-tetrahydropyridine (MPTP)-mediated SH-SY5Y cell toxicity. bold text denotes the highest activity that maximally alleviates cell damage within the compound group.

Compound	MPTP 3.45 mM	EC50 (μM)	Cell Viabilities Under Different Compound Concentrations								
			0 μM	0.78125 μM	1.5625 μM	3.125 μM	6.25 μM	12.5 μM	25 μM	50 μM	100 μM
**1**	-	-	1.00	0.96 ± 0.03	0.95 ± 0.01	0.90 ± 0.04	0.87 ± 0.03	0.88 ± 0.04	0.84 ± 0.05	0.75 ± 0.03	0.42 ± 0.06
	+	inactive	0.61 ± 0.02	0.62 ± 0.02	0.59 ± 0.04	0.60 ± 0.01	0.60 ± 0.04	0.56 ± 0.06	0.55 ± 0.01	0.52 ± 0.03	0.46 ± 0.08
**2**	-	-	1.00	0.98 ± 0.04	0.98 ± 0.01	0.92 ± 0.03	0.91 ± 0.01	0.84 ± 0.05	0.85 ± 0.02	0.79 ± 0.04	0.36 ± 0.03
	+	inactive	0.59 ± 0.02	0.56 ± 0.03	0.55 ± 0.05	0.57 ± 0.04	0.53 ± 0.02	0.50 ± 0.07	0.51 ± 0.04	0.51 ± 0.02	0.43 ± 0.02
**3**	-	-	1.00	0.98 ± 0.02	0.96 ± 0.05	0.88 ± 0.03	0.89 ± 0.06	0.85 ± 0.04	0.84 ± 0.09	0.80 ± 0.07	0.34 ± 0.02
	+	inactive	0.63 ± 0.05	0.62 ± 0.02	0.60 ± 0.05	0.60 ± 0.06	0.56 ± 0.08	0.59 ± 0.04	0.56 ± 0.03	0.57 ± 0.03	0.44 ± 0.03
**4**	-	-	1.00	1.03 ± 0.03	1.09 ± 0.04	1.00 ± 0.04	0.99 ± 0.09	0.98 ± 0.11	0.93 ± 0.03	0.95 ± 0.08	0.89 ± 0.07
	+	0.52	0.52 ± 0.05	0.61 ± 0.09	0.61 ± 0.05	**0.64 ± 0.03**	0.59 ± 0.01	0.60 ± 0.01	0.49 ± 0.03	0.45 ± 0.03	0.44 ± 0.04
**5**	-	-	1.00	1.13 ± 0.08	1.08 ± 0.05	0.94 ± 0.06	1.02 ± 0.06	0.99 ± 0.07	1.01 ± 0.05	0.99 ± 0.07	0.94 ± 0.06
	+	0.39	0.58 ± 0.11	**0.87 ± 0.06**	0.78 ± 0.04	0.77 ± 0.10	0.67 ± 0.12	0.63 ± 0.07	0.48 ± 0.07	0.53 ± 0.09	0.47 ± 0.08
**6**	-	-	1.00	1.12 ± 0.08	1.10 ± 0.12	1.04 ± 0.05	1.05 ± 0.11	0.96 ± 0.06	0.93 ± 0.06	0.94 ± 0.05	0.94 ± 0.06
	+	0.39	0.49 ± 0.06	**0.63 ± 0.05**	0.61 ± 0.04	0.59 ± 0.05	0.53 ± 0.05	0.52 ± 0.06	0.47 ± 0.04	0.40 ± 0.03	0.33 ± 0.06
**7**	-	-	1.00	0.90 ± 0.06	0.87 ± 0.03	0.89 ± 0.06	0.88 ± 0.03	0.90 ± 0.03	0.92 ± 0.05	0.92 ± 0.06	0.70 ± 0.03
	+	0.39	0.63 ± 0.04	**0.71 ± 0.05**	0.61 ± 0.09	0.60 ± 0.08	0.56 ± 0.06	0.61 ± 0.09	0.65 ± 0.02	0.60 ± 0.04	0.59 ± 0.02
**8**	-	-	1.00	1.04 ± 0.04	0.97 ± 0.03	0.97 ± 0.06	0.95 ± 0.003	0.93 ± 0.05	0.93 ± 0.02	0.92 ± 0.06	0.88 ± 0.02
	**+**	inactive	0.48 ± 0.01	0.45 ± 0.02	0.38 ± 0.07	0.36 ± 0.04	0.27 ± 0.05	0.31 ± 0.02	0.16 ± 0.02	0.04 ± 0.01	0.03 ± 0.01

**Figure A10:**
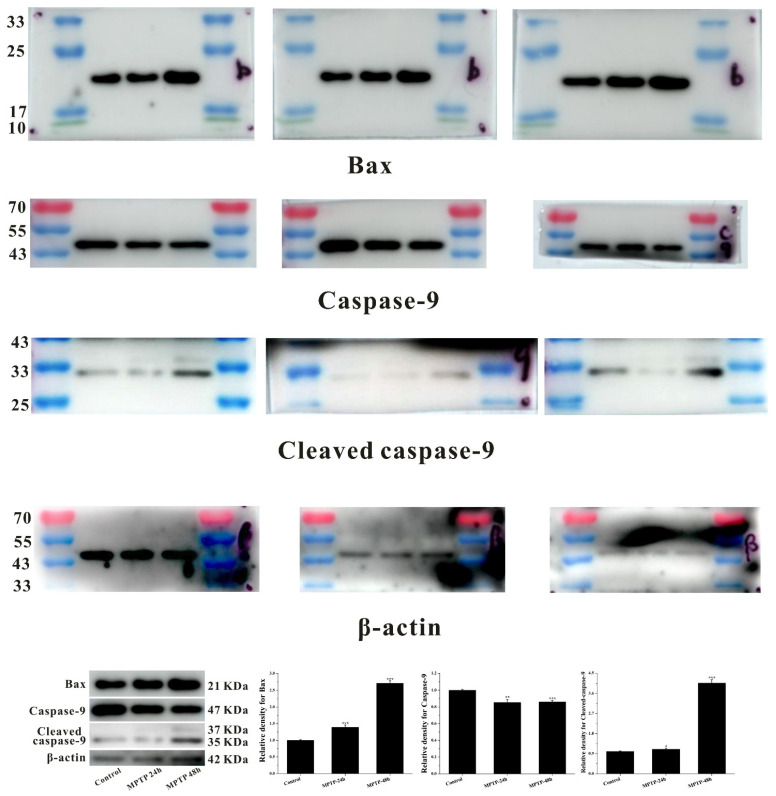
The original WB bands correspond to Bax, Caspase-9, and Cleaved caspase-9 for three repeats. * *p* < 0.05, ** *p* < 0.01, *** *p* < 0.001.

**Figure A11:**
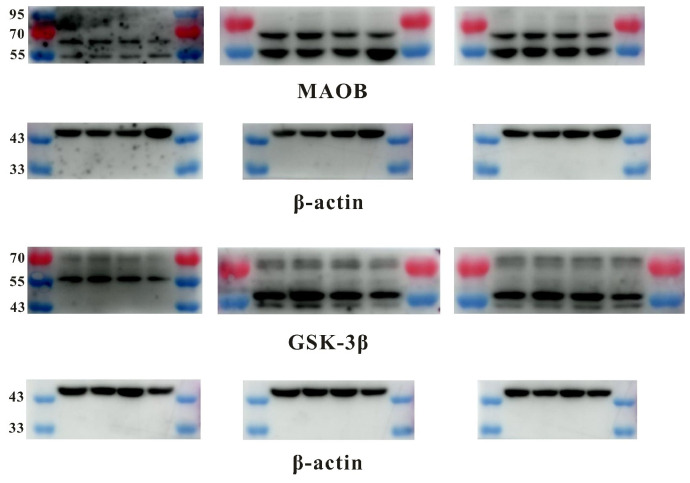

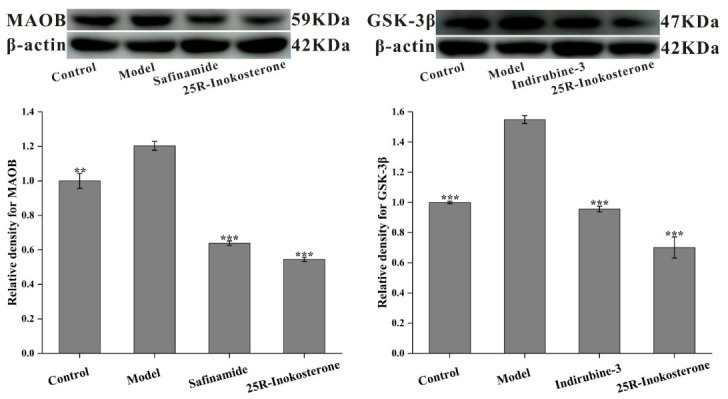
The original WB bands correspond to MAOB and GSK-3β for three repeats. ** *p* < 0.01, *** *p* < 0.001.

**Figure A12:**
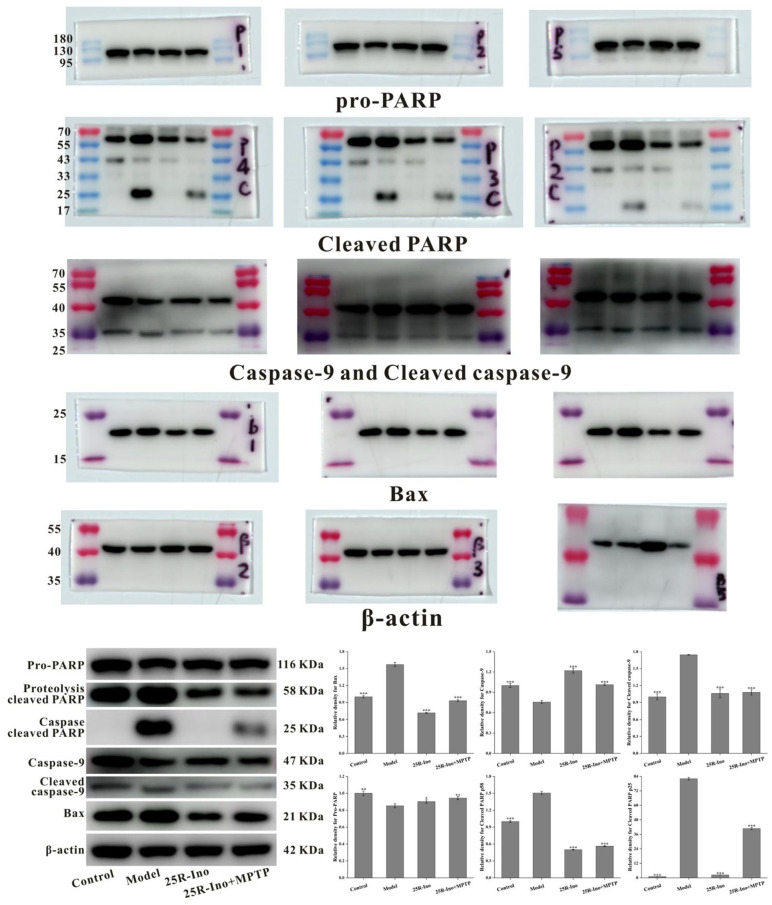
The original WB bands correspond to pro-PARP, Cleaved PARP, Caspase-9, Cleaved caspase-9, and Bax for three repeats. * *p* < 0.05, ** *p* < 0.01, *** *p* < 0.001.

**Figure A13:**
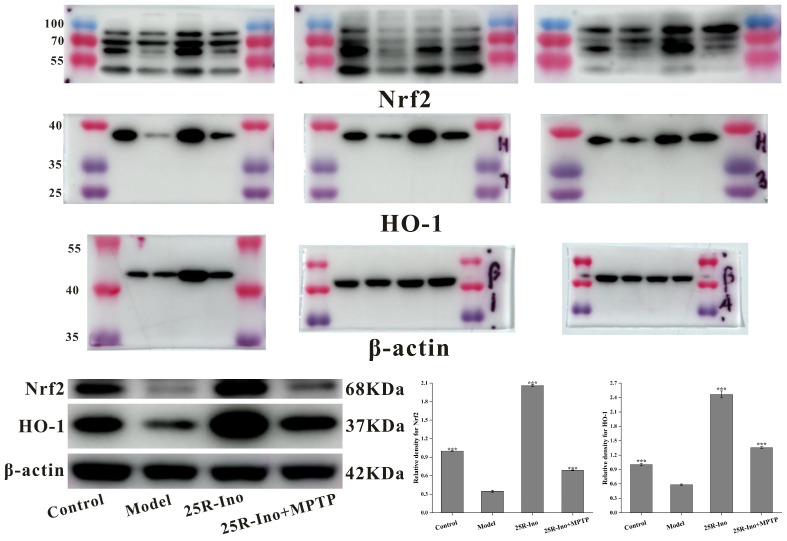
The original WB bands correspond to Nrf2 and HO-1 for three repeats. *** *p* < 0.001.

**Figure A14:**
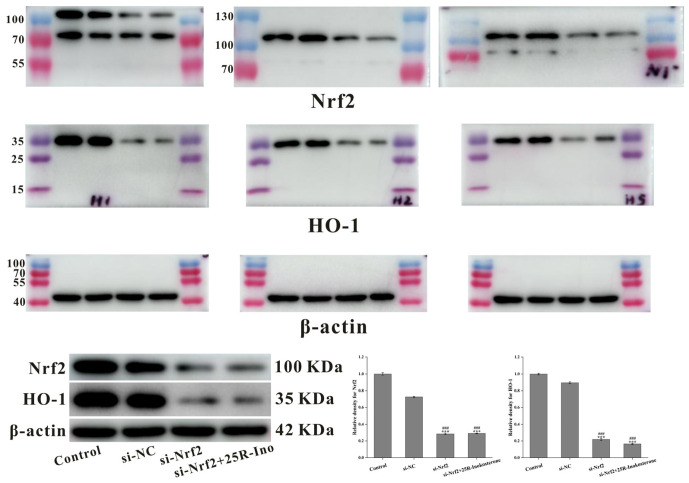
The original WB bands correspond to MAOB and GSK-3β (treated with siRNA) for three repeats. *** *p* < 0.001; ### *p* < 0.001.

**Figure A15:**
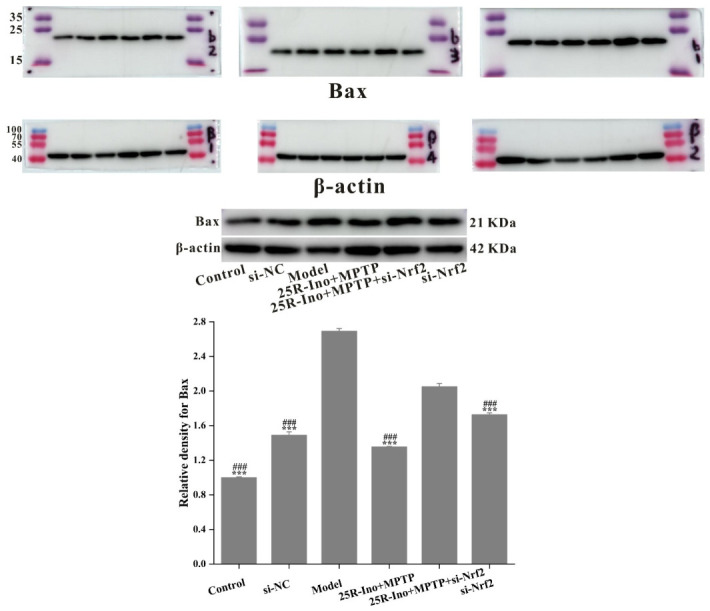
The original WB bands correspond to Bax (treated with siRNA) for three repeats. *** *p* < 0.001; ### *p* < 0.001.

**Figure A16:**
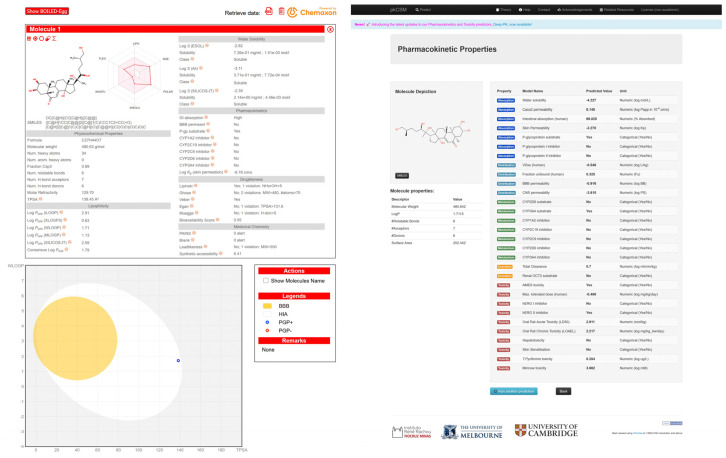
In silico ADME and pharmacokinetic predictions of 25*R*-inokosterone from SwissADME and pkCSM.

## Data Availability

The original contributions presented in this study are included in the article. Further inquiries can be directed to the corresponding author.

## Abbreviations

The following abbreviations are used in this manuscript:

PD	Parkinson’s disease
AD	Alzheimer’s disease
CNS	Central nervous system
PNS	Peripheral nervous system
MAOB	Monoamine oxidase B
GSK-3β	Glycogen synthase kinase-3β
DAT	Dopamine transporter
SNCA	α-Synuclein
Nrf2	Nuclear factor erythroid 2-related factor 2
HO-1	Heme oxygenase-1
MMP	Mitochondrial membrane potential
ΔΨm	Mitochondrial transmembrane potential
ROS	Reactive oxygen species
MPTP	1-Methyl-4-phenyl-1,2,3,6-tetrahydropyridine
MPP^+^	1-Methyl-4-phenylpyridinium
HPLC	High-performance liquid chromatography
NMR	Nuclear magnetic resonance
TOF-MS	Time-of-flight mass spectrometry
HSQC	Heteronuclear single quantum coherence
HMBC	Heteronuclear multiple bond correlation
TMS	Tetramethylsilane
TLC	Thin-layer chromatography
DMEM/F12	Dulbecco’s Modified Eagle Medium/Nutrient Mixture F-12
FBS	Fetal bovine serum
PMSF	Phenylmethanesulfonyl fluoride
BCA	Bicinchoninic acid
ECL	Enhanced chemiluminescence
ELISA	Enzyme-linked immunosorbent assay
GSH	Glutathione
LDH	Lactate dehydrogenase
siRNA	Small interfering RNA
si-NC	Small interfering RNA negative control
si-Nrf2	Small interfering RNA targeting Nrf2
HRP	Horseradish peroxidase
TBST	Tris-buffered saline with Tween-20
BSA	Bovine serum albumin
PFA	Paraformaldehyde
TUNEL	Terminal deoxynucleotidyl transferase dUTP nick end labeling
DCFH-DA	2′,7′-Dichlorodihydrofluorescein diacetate
FITC	Fluorescein isothiocyanate
PI	Propidium iodide
PPI	Protein–protein interaction
GO	Gene Ontology
KEGG	Kyoto Encyclopedia of Genes and Genomes
DAVID	Database for Annotation, Visualization and Integrated Discovery
STRING	Search Tool for the Retrieval of Interacting Genes/Proteins
PDB	Protein Data Bank
DEGs	Differentially expressed genes
EC_50_	Half maximal effective concentration
ANOVA	Analysis of variance
SD	Standard deviation
Aβ	β-Amyloid
ER	Endoplasmic reticulum
RMSD	Root-mean-square deviation

## Appendix A

**Table A1:** Training and testing set molecules for constructing a three-dimensional pharmacophore model for virtual screening of MAOB and GSK-3 β expression modulators.

Targets	Structures	
GSK-3β	BRD0209 (Training set)	BRD3937 (Training set)
	PF-04802367 (Training set)	BRD0705 (Training set)
	C22 (Training set)	Inhibitor 16 (Training set)
	Ligand 1 (Training set)	BRD3731 (Training set)
	BI-91BS (Training set)	PIK-75 (Training set)
GSK-3β	CHIR99021 (Test set)	PDB 5OY4 (Test set)
	C50 (Test set)	PDB 5T31 (Test set)
MAOB	N-(3-chlorophenyl)-4-oxo-4H-chromene-3-carboxamide (Training set)	PF9601N (Test set)
	Pioglitazone (Training set)	RG0216 (Test set)
	Safinamide (Training set)	Ladostigil_(Test set)
